# Comparative Analysis of Transcriptomic and Proteomic Expression between Two Non-Small Cell Lung Cancer Subtypes

**DOI:** 10.1021/acs.jproteome.4c00773

**Published:** 2025-01-08

**Authors:** Ben Nicholas, Alistair Bailey, Katy J. McCann, Peter Johnson, Tim Elliott, Christian Ottensmeier, Paul Skipp

**Affiliations:** † Centre for Proteomic Research, School of Biological Sciences and Institute for Life Sciences, University of Southampton, Building 85, Southampton SO17 1BJ ,U.K.; ‡ Centre for Cancer Immunology and Institute for Life Sciences, Faculty of Medicine, University of Southampton, Southampton SO16 6YD ,U.K.; § School of Cancer Sciences, Faculty of Medicine, University of Southampton, Southampton SO16 6YD ,U.K.; ∥ Cancer Research UK Clinical Centre, University of Southampton, Southampton SO16 6YD ,U.K.; ⊥ Oxford Cancer Centre for Immuno-Oncology and CAMS-Oxford Institute, Nuffield Department of Medicine, University of Oxford, Oxford OX3 7LE ,U.K.; # Institute of Systems, Molecular and Integrative Biology, University of Liverpool, Liverpool L69 7BE, U.K.

**Keywords:** proteomics, gene expression, non-small cell lung cancer

## Abstract

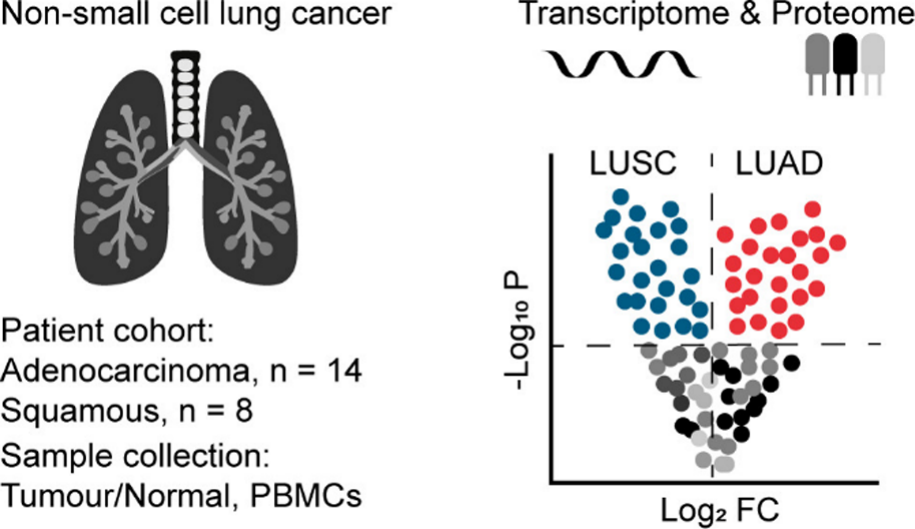

Non-small cell lung cancer (NSCLC) is frequently diagnosed late and has poor survival. The two predominant subtypes of NSCLC, adenocarcinoma (LUAD) and squamous cell carcinoma (LUSC), are currently differentially diagnosed using immunohistochemical markers; however, they are increasingly recognized as very different cancer types suggestive of potential for new, more targeted therapies. There are extensive efforts to find more precise and noninvasive differential diagnostic tools. Here, we examined these two NSCLC subtypes for differences that may inform treatment and identify potential novel therapeutic pathways. We presented a comparative analysis of transcriptomic and proteomic expression in tumors from a cohort of 22 NSCLC patients: 8 LUSC and 14 LUAD. Comparing NSCLC subtypes, we found differential gene expression related to cell differentiation for LUSC and cellular structure and immune response regulation for LUAD. Differential protein expression between NSCLC subtypes was related to extracellular structure for LUSC and metabolic processes, including glucose metabolism for LUAD. This direct comparison was more informative about subtype-specific pathways than between each subtype and control (nontumor) tissues. Many of our observations between NSCLC subtypes support and inform existing observations and reveal differences that may aid research seeking to identify and validate novel subtype biomarkers or druggable targets.

## Introduction

Lung cancer is the second most common cancer in the UK, with a majority of cases diagnosed at advanced stages, either locally advanced (stage III) or metastatic (stage IV). Non-small cell lung cancer (NSCLC) comprises 85–90% of these cases and is further categorized into three histological subtypes: adenocarcinoma (LUAD), the most common type, typically develops in the alveoli of the outer peripheral lung; squamous cell carcinoma (LUSC), the second most frequent type, usually forms in squamous cells located more centrally in the lungs; and large cell undifferentiated carcinoma, the least common, can originate anywhere in the lung.^1^ In the UK, less than 20% of all lung cancer patients survive for 5 years, with the majority of patients surviving less than one year post-diagnosis.^2,3^ While NSCLC subtypes are usually differentially diagnosed using histochemical markers, recognizing proteins such as napsin-A (NAPSA), homeobox protein Nkx-2.1 (TTF1), tumor protein 63 (TP63) and cytokeratins 5/6 (K2C5/6A), any refinement in the selection of these biomarkers is welcome, and the impacts on therapeutic options remain limited. However, it is increasingly recognized that LUAD and LUSC are very different cancer types with unique clinical features,^4^ and there may be potential for tailor-made chemotherapeutic or immunotherapeutic options targeted at each type.

Consequently, there are extensive efforts to find more precise and noninvasive diagnostics for NSCLC, for example, using circulating proteins^5^ or miRNAs,^6^ which might also inform novel therapeutic pathways. Likewise, the longitudinal NSCLC TRACERx (TRAcking Cancer Evolution through therapy (Rx)) study has sought to identify the evolutionary processes that help explain disease progression and treatment resistance.^7^ Furthermore, we have previously presented identification of HLA-presented neoantigens as cancer vaccine targets in two NSCLC subtypes, squamous cell carcinoma (LUSC) and adenocarcinoma (LUAD).^8^

Here, we present a comparative analysis of transcriptomic and proteomic expression in tumors from a cohort of 22 NSCLC patients: 8 LUSC and 14 LUAD. The patients were from the same cohort as for our neoantigen study. Using RNA sequencing (RNAseq) and label-free quantification (LFQ) of bottom-up mass spectrometry proteomics, we sought to identify differences that may inform treatment options and therapeutic pathways. NSCLC subtype transcriptomes were compared to each other and to peripheral blood mononuclear cells (PBMCs). NSCLC-subtype proteomes were also compared to each other and to normal adjacent lung tissue (NAT).

Using differential expression analysis, we identified genes and proteins that characterize each NSCLC subtype.

Our observations offer independent corroboration and contrast to existing studies to aid further research to identify NSCLC subtype biomarkers or targets for more effective subtype-specific treatments.

## Materials and Methods

### Ethics Statement

Ethical approval was obtained from the local research ethics committee (LREC reference 14-SC-0186 150975), and written informed consent was provided by the patients.

### Tissue Preparation

Tumors were excised from resected lung tissue postoperatively by pathologists and processed either for histological evaluation of tumor type and stage or snap-frozen at −80 °C. Whole blood samples were obtained, and PBMCs were isolated by density gradient centrifugation over Lymphoprep prior to storage at −80 °C.

### RNA Extraction

RNA was extracted from tumor tissue that had been obtained fresh and immediately snap-frozen in liquid nitrogen. Ten to twenty 10 μm cryosections were used for nucleic acid extraction using an automated Maxwell RSC instrument (Promega) and a Maxwell RSC simplyRNA tissue kit according to the manufacturer’s instructions. RNA was quantified using the Qubit fluorometric quantitation assay (ThermoFisher Scientific) according to the manufacturer’s instructions. RNA quality was assessed using an Agilent 2100 Bioanalyzer to generate an RNA integrity number (RIN; Agilent Technologies UK Ltd.).

### RNA Sequencing

Samples were prepared as TruSeq-stranded mRNA libraries (Illumina, San Diego, USA), and 100 bp paired-end sequencing was performed using the Illumina NovaSeq 6000 system by Edinburgh Genomics (Edinburgh, UK). Raw reads were preprocessed using fastp (version 0.20.0).^9^

Filtered reads were aligned twice: First, to the 1000 genomes project version of the human genome reference sequence (GRCh38/hg38) using HISAT2 (version 2.2.1),^10^ the reads were merged and then transcripts assembled, and gene expression was estimated with featureCounts (version 2.0.6)^11^ using reference-guided assembly. Second, reads were aligned and quantified using transcript classification with Salmon (version 1.10.3).^12^

### Differential Gene Expression

Differentially expressed genes (DEGs) were estimated using transcript counts from both HISAT2 and Salmon with edgeR (version 4.0.2) using default settings.^13^ The intersection of DEGs common to both the HISAT2 and Salmon analyses was used to filter the HISAT2 results that were used for the remaining analysis.

Principal component analysis (PCA) of the normalized HISAT2 count matrices was performed using DESEq2 (version 1.44.0)^14^ and PCATools (version 2.16.0).^15^

Results were visualized using EnhancedVolcano (version 1.22.0),^16^ pheatmap (version 1.0.12),^17^ and ggplot2 (version 3.5.1).^18^

### Protein Extraction and Digestion

Snap-frozen tissue samples were briefly thawed and weighed prior to 30 s of mechanical homogenization (Fisherbrand homogenizer 150 using plastic generator probes, Fisher Scientific, UK) in 8 mL of lysis buffer (0.02 M Tris, 0.5% (w/v) IGEPAL, 0.25% (w/v) sodium deoxycholate, 0.15 mM NaCl, 1 mM EDTA, 0.2 mM iodoacetamide supplemented with EDTA-free protease inhibitor mix) and incubated at 4 °C for 30 min. Homogenates were then centrifuged at 2000*g* for 10 min at 4 °C to remove cell debris and for a further 60 min at 13,000*g*, 4 °C, for clarification. The supernatant was stored at −80 °C prior to protein extraction for proteomic analysis.

Protein concentration of tissue lysates was determined by BCA assay, and volumes equivalent to 100 μg of protein were precipitated using methanol/chloroform, as previously described.^19^ Pellets were briefly air-dried prior to resuspension in 6 M urea/50 mM Tris-HCl (pH 8.0). Proteins were reduced by the addition of 5 mM (final concentration) DTT and incubated at 37 °C for 30 min, then alkylated by the addition of 15 mM (final concentration) iodoacetamide, and incubated in the dark for 30 min. Four micrograms of Trypsin/LysC mix (Promega) was added, and the sample was incubated for 4 h at 37 °C, then 6 volumes of 50 mM Tris-HCl pH 8.0 were added to dilute the urea to <1 M, and the sample was incubated for a further 16 h at 37 °C. Digestion was terminated by the addition of 4 μL of TFA, and the sample was clarified at 13,000*g* for 10 min at RT. The supernatant was collected and applied to Oasis Prime microelution HLB 96-well plates (Waters, UK), which had been pre-equilibrated with acetonitrile. Peptides were eluted with 50 μL of 70% acetonitrile and dried by vacuum centrifugation prior to resuspension in 0.1% formic acid.

### Mass Spectrometry Proteomics

Eight micrograms of peptides per sample was separated by an Ultimate 3000 RSLC nanosystem (Thermo Scientific) using a PepMap C18 EASY-Spray LC column, 2 μm particle size, 75 μm × 75 cm column (Thermo Scientific) in buffer A (H_2_O/0.1% Formic acid) and coupled online to an Orbitrap Fusion Tribrid Mass Spectrometer (Thermo Fisher Scientific, UK) with a nanoelectrospray ion source.

Peptides were eluted with a linear gradient of 3–30% buffer B (acetonitrile/0.1% formic acid) at a flow rate of 300 μL/min over 200 min. Full scans were acquired in the Orbitrap analyzer in the scan range of 300–1500 *m*/*z* using the top speed data-dependent mode, performing an MS scan every 3 s cycle, followed by higher energy collision-induced dissociation (HCD) MS/MS scans. MS spectra were acquired at a resolution of 120,000, an RF lens of 60%, and an automatic gain control (AGC) ion target value of 4.0e5 for a maximum of 100 ms. MS/MS scans were performed in the ion trap, and higher energy collisional dissociation (HCD) fragmentation was induced at an energy setting of 32% and an AGC ion target value of 5.0e3.

### Proteomics Data Analysis

Raw spectrum files were analyzed using Peaks Studio 10.0 build 20190129,^20,21^ and the data were processed to generate reduced charge state and deisotoped precursor and associated product ion peak lists, which were searched against the UniProt database (20,350 entries, 2020-04-07) plus the corresponding mutanome for each sample (∼1000–5000 sequences) and a contaminant list in unspecific digest mode. Parent mass error tolerance was set to 10 ppm, and fragment mass error tolerance was set to 0.6 Da. Variable modifications were set for N-term acetylation (42.01 Da), methionine oxidation (15.99 Da), and carboxyamidomethylation (57.02 Da) of cysteine. A maximum of three variable modifications per peptide was set. The false discovery rate (FDR) was estimated with decoy-fusion database searches^20^ and filtered to 1% FDR.

### Differential Protein Expression

LFQ was performed using the Peaks Q module of Peaks Studio,^20,22^ yielding matrices of protein identifications as quantified by their normalized top 3 peptide intensities. The resulting matrices were filtered to remove any proteins for which there were more than two missing values across the samples. Differential protein expression was then calculated with DEqMS using the default parameters.^23^

PCA of the normalized top 3 peptide intensities was performed using DESEq2^14^ and PCATools.^15^

Results were visualized using EnhancedVolcano,^16^ pheatmap,^17^ and ggplot2.^18^

### Functional Analysis

Functional enrichment analysis was performed using g:Profiler^24^ with default settings for *Homo sapiens* modified to exclude GO electronic annotations. Gene identifiers were used as inputs for DEGs and protein identifiers for DEPs.

## Results

### NSCLC Patient Cohort

Table 1 summarizes our cohort of 22 NSCLC patients with either LUSC (*n* = 8) or LUAD (*n* = 14) subtype. Tumor tissues underwent RNaseq and mass spectrometry proteomics (LFQ). Whole exome sequencing was used to calculate tumor purity and ploidy.^8,25,26^

**Table 1:** Summary of Patients in This Study with Non-Small Cell Lung Cancer

donor	cancer subtype	smoking status	tumor wet weight (mg)	tumor purity	tumor ploidy
A113	LUSC	current smoker	26	1.0	2.0
A115	LUSC	ex smoker	129	0.5	1.9
A116	LUSC	never smoker	113	0.5	2.5
A133	LUSC	ex smoker	85	1.0	4.3
A134	LUSC	current smoker	152	0.5	1.8
A140	LUSC	ex smoker	71	0.4	2.7
A144	LUSC	ex smoker	122	0.5	3.8
A152	LUSC	ex smoker	288	0.3	4.7
A114	LUAD	ex smoker	1,085	1.0	2.0
A117	LUAD	current smoker	158	0.3	1.9
A118	LUAD	ex smoker	132	1.0	2.0
A120	LUAD	ex smoker	18	1.0	2.0
A136	LUAD	never smoker	98	0.3	2.0
A137	LUAD	ex smoker	270	1.0	2.0
A139	LUAD	ex smoker	93	0.4	1.6
A141	LUAD	ex smoker	391	0.3	5.2
A142	LUAD	current smoker	277	1.0	2.6
A143	LUAD	ex smoker	97	0.5	1.9
A146	LUAD	current smoker	99	1.0	2.0
A147	LUAD	current smoker	88	1.0	2.2
A148	LUAD	ex smoker	229	1.0	2.0
A153	LUAD	ex smoker	436	0.4	2.6

PBMCs were available for RNaseq for 10 of the LUAD patients and 5 of the LUSC patients; 9 LUAD and 5 LUSC patients had NAT available for proteomics analysis (Table 2).

**Table 2:** Summary of Sample Availability for Patients in This Study with Non-Small Cell Lung Cancer

donor	cancer subtype	tumor RNaseq	PBMC RNaseq^a^	tumor proteome	NAT proteome^b^
A113	LUSC	yes	no	yes	no
A115	LUSC	yes	no	yes	no
A116	LUSC	yes	no	yes	no
A133	LUSC	yes	yes	yes	yes
A134	LUSC	yes	yes	yes	yes
A140	LUSC	yes	yes	yes	yes
A144	LUSC	yes	yes	yes	yes
A152	LUSC	yes	yes	yes	yes
A114	LUAD	yes	no	yes	no
A117	LUAD	yes	no	yes	no
A118	LUAD	yes	no	yes	no
A120	LUAD	yes	no	yes	no
A136	LUAD	yes	yes	yes	yes
A137	LUAD	yes	yes	yes	yes
A139	LUAD	yes	yes	yes	yes
A141	LUAD	yes	yes	yes	yes
A142	LUAD	yes	yes	yes	no
A143	LUAD	yes	yes	yes	yes
A146	LUAD	yes	yes	yes	yes
A147	LUAD	yes	yes	yes	yes
A148	LUAD	yes	yes	yes	yes
A153	LUAD	yes	yes	yes	yes

a Peripheral blood mononuclear cells.

b Normal adjacent tissue.

Although it is not technically correct to describe genes or proteins as expressed, transcripts are expressed, and proteins are the products of translation, the word expression has become synonymous for the product of a biological process. Hence, we here refer throughout to the quantification of transcripts and peptides as gene expression and protein expression, respectively.^27,28^

### Principal Component Analysis Comparison of NSCLC Transcriptomes and Proteomes

For the transcriptomes, count matrices for each sample were calculated containing gene expression values, as represented by transcript abundance counts for each gene. One matrix was calculated from genomic alignments and feature counting^10,11^ and a second matrix from transcript classification^12^ (Tables S1–S6). For the proteomes, proteins were quantified using LFQ,^20,22^ yielding protein identifications from the normalized top 3 peptide intensities (Tables S10–S12).

**Figure 1:**
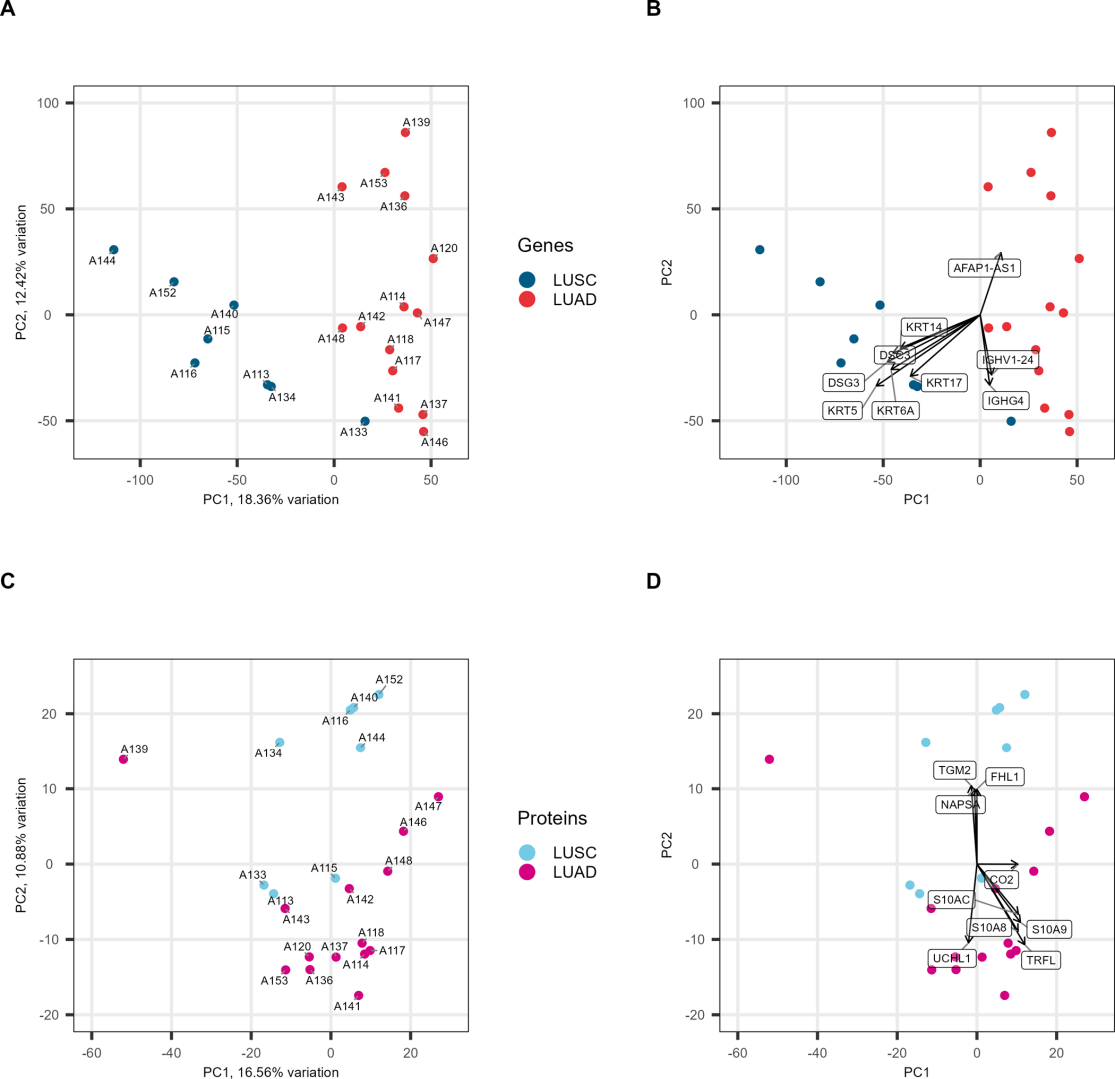
Biplots of the LUSC and LUAD subtype comparison. (A) PCA of the normalized gene count matrix numbered with donor identifier. LUSC (blue) and LUAD (red). (B) PCA of the normalized gene count matrix with the genes contributing to the PC directions annotated. (C) PCA of the normalized top 3 peptide intensities numbered with donor identifier. LUSC (light blue) and LUAD (purple). (D) PCA of the normalized top 3 peptide intensities with the protein contributing to the PC directions annotated.

To examine how well NSCLC subtypes cluster, assess within-group similarity, and identify batch effects or outlier individuals, we performed PCA using the normalized feature count data for the transcriptomes and the normalized top 3 peptide intensities for the proteomes^14,15^ (Figures 1, S1, and S2).

Gene expression comparison between LUSC and LUAD found that the two cancer subtypes divide along PC1; however, there are clusters within each subtype and some individuals, notably LUSC individuals A144 and A133 (Figure 1A). There were no obvious batch effects. The first two PCs account for 30% of the variance. Gene expressions contributing most to the PC1 separation between LUSC and LUAD are keratins and cadherins, such as keratin 5 (KRT5), desmoglein-3 (DSG-3), and desmocollin-3 (DSC3). The expression of long non-coding RNA (lncRNA) actin filament-associated protein 1 antisense RNA 1 (AFAP1-AS1) drives the separation of LUAD individuals A143, A153, A139, and A136 along PC2. The expression of immunoglobulin heavy constant gamma 4 (IGHG4) and immunoglobulin heavy variable 1–24 (IGHV1–24) contributes to separation along PC2 in the other direction (Figure 1B).

Protein expression showed a less clear separation between LUSC and LUAD samples. PCs 1 and 2 account for 28% of the variance, and without the labels, the division between them would not be obvious (Figure 1C). LUAD patient A139 is an outlier, while LUSC patients A113, A115, and A133 sit close to the majority of LUAD samples. The separation between LUSC and LUAD is primarily along PC2 and toward LUSC separation driven by protein-glutamine gamma-glutamyltransferase 2 (TGM2), napsin-A (NAPSA), and four and a half LIM domains protein 1 (FHL1). Toward LUAD along PC2 are proinflammatory metal-binding proteins, protein S100-A8 (S10A8), protein S100-A9 (S10A9), and protein S100-A12 (S10AC), lactotransferrin (TRFL), and ubiquitin carboxyl-terminal hydrolase (UCHL1) (Figure 1D).

Comparison of NSCLC subtypes with PBMC for gene expression and NAT for protein expression showed a clear separation between tumor and non-tumor samples along PC1 and individual sample variation along PC2 (Figures S1 and S2). For LUSC and LUAD, gene expression of collagens and keratins drove the separation of tumor tissue from PBMC. Differences in protein expression of hemoglobin subunits A and B (HBA, HBB) and advanced glycosylation end product-specific receptor drove the separation of LUSC and LUAD tumor tissues from NAT (Figures S1 and S2).

### Differential Gene and Protein Expression in NSCLC

For all 22 NSCLC patients, we calculated differential gene expression (DEG) and differential protein expression (DEP) between LUSC and LUAD. We calculated DEG between LUSC and PBMC (*n* = 5) and DEP between LUSC and NAT (*n* = 5). Likewise, we calculated DEG between LUAD and PBMC (*n* = 10) and DEP between LUAD and NAT (*n* = 9).

Using the transcriptomes, samples were grouped according to LUSC or LUAD subtype or PBMC, and DEGs were calculated from both genomic alignment and transcript classification count matrices using edgeR.^13^ Each final DEG table was filtered for genes common to both analyses (Table 3). DEGs for shared genes from genomic alignments with HISAT2 are shown here, and the transcript classification results from Salmon are provided in the Supporting Information.

**Table 3:** Comparison of the DEGs

comparison	total DEGs	LUSC DEGs^a^	LUAD DEGs^a^
LUSC and PBMCs	17,719	3989	
LUAD and PBMCs	17,586		3822
LUSC and LUAD	19,859	265	51

a DEG above thresholds of a log2 fold-change of 1.5 and an FDR of 1%.

Table 3 shows the numbers of DEGs for each NSCLC subtype comparison exceeding thresholds of a log_2_ fold-change of 1.5 and below a false discovery rate (FDR) of 1%. These thresholds are necessarily arbitrary and chosen to balance being conservative while not overexcluding information. The data without thresholds are provided in Supporting Information Tables S7–S9.

Using the proteomes quantified using the normalized top 3 peptide intensities, samples were grouped according to LUSC or LUAD subtype or NAT, and DEP was calculated by DEqMS.^23^ Mass spectrometry proteomics quantifies far fewer proteins than transcriptomes do due to methodological differences. For any protein (or gene) to be analyzed for differential expression, it must be present in all samples under consideration. The total number of DEPs quantified for NSCLC and NAT comparisons is approximately one-third of the DEPs quantified comparing LUSC and LUAD (Table 4). For DEPs, we used more relaxed thresholds to filter the results than those for the DEGs of a log_2_ fold-change of 1 and a significance below a *p*-value of 0.01. The data without thresholds are provided in the Supporting Information Tables S13–S15.

**Table 4:** Comparison of DEPs

comparison	total DEPs	LUSC DEPs^a^	LUAD DEPs^a^
LUSC and NAT	1330	326	
LUAD and NAT	1478		185
LUSC and LUAD	3872	117	15

a DEP above thresholds of log2 fold-change of 1 and a *p*-value of 1%.

**Figure 2:**
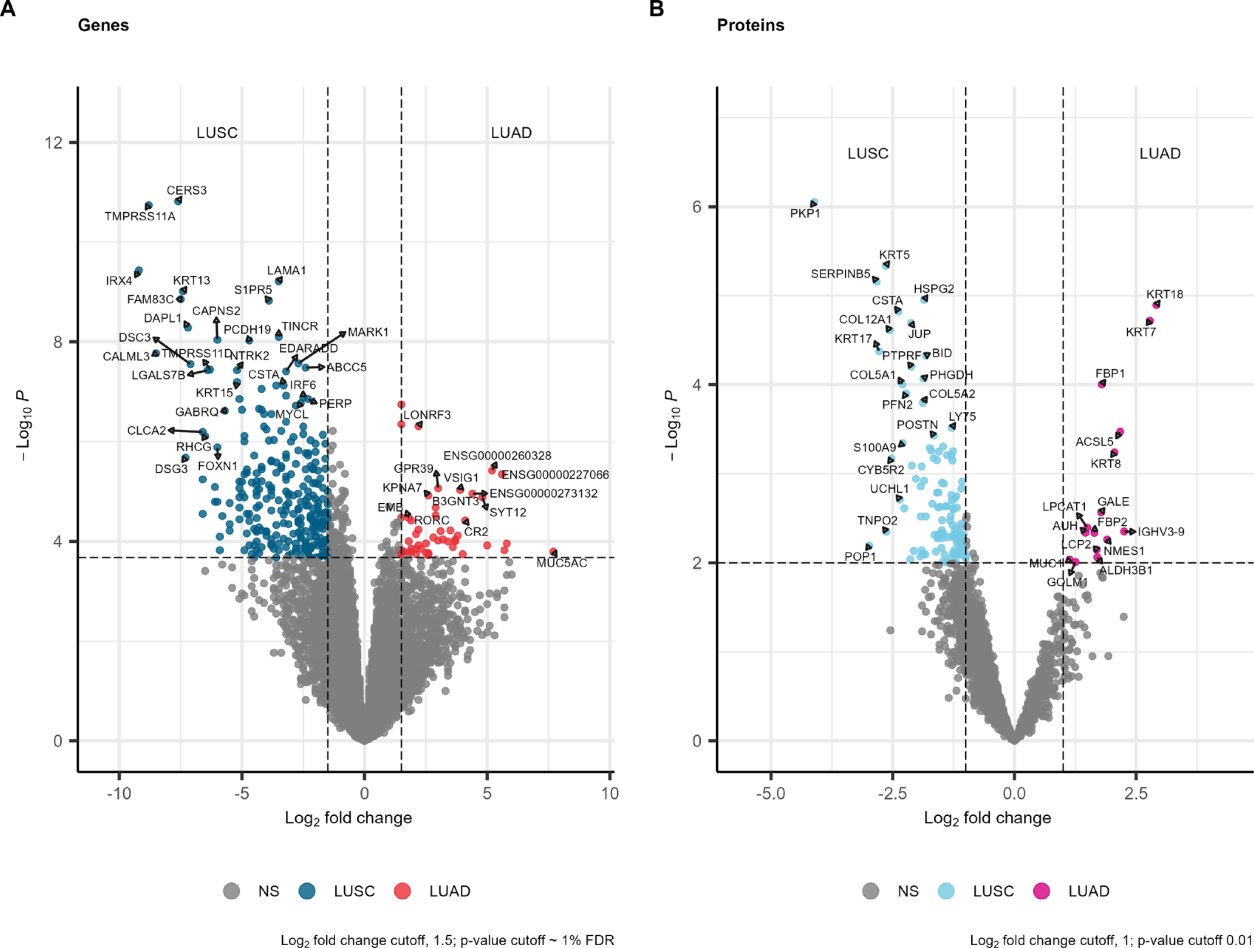
Volcano plots of DEGs and DEPs. Gene names are used on both plots. (A) Comparison of LUSC and LUAD genes (*n* = 19,859). Thresholds are represented by dotted lines at an FDR of 1% and log_2_ fold change of 1.5. NS is any DEG below these thresholds. (B) Comparison of LUSC and LUAD proteins (*n* = 3872). Thresholds are represented by dotted lines at a *p*-value of 1% and a log_2_ fold change of 1. NS is any DEP below these thresholds.

Comparing NSCLC subtypes, only 316 of 19,859 DEGs exceeded the thresholds of a log_2_ fold-change of 1.5 and an FDR below 1%. Of these 316 genes, 265 were enriched in LUSC and 51 in LUAD (Table 3 and Figure 2A). LUAD had two highly expressed novel transcript lncRNAs, ENSG00000227066 and ENSG00000260328, and an antisense lncRNA, ENSG00000273132. ENSG00000273132 is antisense to LDL receptor-related protein 11 (LRP11).

Differential expression of proteins between NSCLC subtypes yielded only 132 of 3872 DEPs exceeding the thresholds of a log_2_ fold-change of 1 and below a *p*-value of 1%. Of these 132 proteins, 117 were enriched in LUSC and 15 in LUAD (Table 4 and Figure 2B).

When comparing NSCLC subtypes to PBMC or NAT, we observed nearly 4000 tumor DEGs and close to 1500 DEPs for each subtype (Tables 3, 4, Figures S3, and S4). These observations support the findings from the PCA that tumor tissues are highly dissimilar to either PBMC or NAT. Likewise, the individual sample variation in DEGs and DEPs highlighted the heterogeneity within NSCLC subtypes observed in the PCA (Figures S5–S7).

## Functional Analysis

Finally, we sought functional interpretations of the genes and proteins yielded from differential expression analysis. We used g:Profiler to perform enrichment analysis and identify functional processes and pathways.^24^ To select the lists, we chose thresholds that selected similar proportions of DEGs and DEPs from each comparison as inputs to g:Profiler.

**Figure 3:**
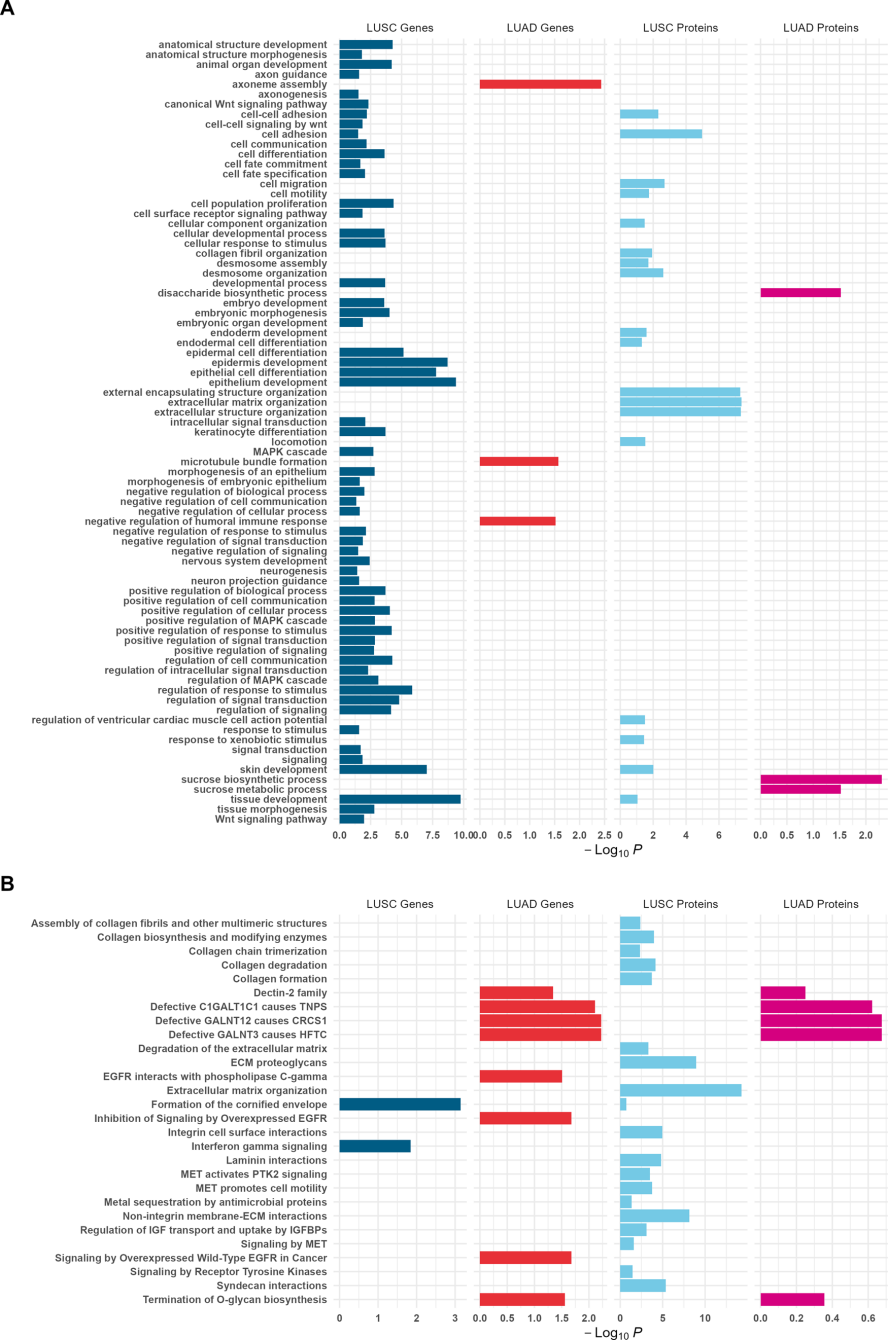
Bar plots of functional enrichment between NSCLC subtypes. Statistical significance level indicated by the −log_10_*p*-value on the *x*-axis. (A) GO biological processes enriched in NSCLC subtypes. (B) Reactome pathways enriched in NSCLC subtypes. Outputs were not filtered, but for reference, a value of −log_10_*P* of 1.3 is equivalent to a *p*-value of 0.05, and a larger −log_10_*P* value equates to a smaller *p*-value and vice versa.

For comparisons between NSCLC subtypes, DEGs were filtered at a log_2_ fold-change of 1.5 and an FDR below 5%, while DEPs were filtered at a log_2_ fold-change of 1 and a *p*-value below 5%. As previously noted, it is worth noting that the unfiltered DEG and DEP lists are provided in Supporting Information Tables S7–S9 and S13–S15 for analysis with alternative thresholds. Likewise, g:Profiler performs enrichment analysis using 11 pathway sources, and the full results are provided in the Supporting Information (Tables S16 and S17). Here, we focused on enrichment of biological processes (GO:BP)^29,30^ and reactome pathways^31^ (Figure 3). We also compared NSCLC subtypes to PBMCs and NAT, filtering DEGs and DEPs at a log_2_ fold-change of 1.5 and an FDR of below 1% (Figure S8). Outputs were not filtered, but for reference, a value of −log_10_*P* of 1.3 is equivalent to a *p*-value of 0.05, and a larger −log_10_*P* value equates to a smaller *p*-value and vice versa.

Gene expression comparison between the NSCLC subtypes indicates many enriched GO:BP terms relating to developmental processes for LUSC, whereas for LUAD, only three GO:BP terms relating to cellular structure, cell motility, and immune evasion were enriched (Figure 3A). Likewise, the protein expression comparison between the NSCLC subtypes indicates many enriched GO:BP terms for LUSC relating to extracellular matrix remodeling and cell migration and motility and only three terms enriched in LUAD relating to metabolic processes, including glucose metabolism (Figure 3A).

GO:BP enrichment for comparisons of the NSCLC subtypes with PBMC indicates similarly enriched pathways for processes, relating to developmental and structural changes for both subtypes. Likewise, comparisons of the NSCLC subtypes with NAT for GO:BP enrichment also identified similarly enriched pathways for both cancer subtypes but this time related to protein translation and RNA-related processing and splicing (Figure S8A).

Enrichment of reactome pathways of DEGs between NSCLC subtypes identified two pathways enriched in LUSC, as for GO:BP, relating to extracellular matrix remodeling and immune system activation (Figure 3B). For LUAD, eight reactome pathways were enriched, and as for GO:BP, they were related to metabolic processes, including altered glycosylation and the immune system (Figure 3B). From DEPs, reactome pathways for LUSC were enriched, relating to collagen formation, extracellular remodeling, and cell motility, whereas LUAD pathways, relating to defective glycosylation, were enriched (Figure 3B).

Enrichment of reactome pathway gene expression for NSCLC subtypes compared to PBMC identified a nearly identical set of pathways for both subtypes, relating to extracellular matrix remodeling and immune system activation (Figure S8A). Likewise, comparisons of the NSCLC subtypes with NAT found reactome pathways corresponding with the GO:BP enrichment we identified, relating to protein synthesis, RNA processing, and mRNA splicing (Figure S8B).

## Discussion

We have previously used a combination of immunopeptidomics and proteogenomics to identify patient-specific neoantigens arising from NSCLC mutations in both LUAD and LUSC in a cohort of current or ex-smokers.^8^ We found the mutational signature in our cohort typical of those in NSCLC in other studies, reflective of tobacco exposure in terms of relatively high mutational burdens and exonic mutations with a predominance of C > A transversions suggestive of excision repair deficiency and C > T transversions, indicating APOBEC cytidine deaminase activity.

Mutations in LUAD driver genes, such as the epidermal growth factor receptor (EGFR) and chromosomal rearrangements involving the ALK tyrosine kinase receptor (ALK), are both prognostic for therapeutic responses^32−35^ and also have actionable therapeutic pathways in the form of tyrosine kinase inhibitors. These mutations are, however, identified in approximately 15 and 5% of LUAD and LUSC cases, respectively, and are thus only useful in a subset of NSCLC.^36^ This subset of NSCLC is predominant in LUAD in never-smokers, suggesting that they represent a distinct disease with separate etiology from lung cancer in smokers.

Outside of these subgroups, despite the ability to differentiate LUAD and LUSC using immunohistochemistry markers, such as napsin-A (NAPSA), homeobox protein Nkx-2.1 (TTF1) for LUAD, and tumor protein 63 (TP63) and cytokeratins 5/6 (K2C5/6A) for LUSC, treatment pathways for LUAD and LUSC are not tailored to each subtype, consisting predominantly of chemotherapy/radiotherapy and surgical intervention. Additionally, all of these markers have variable sensitivity and specificity for each NSCLC subtype, and their specificity can be negatively impacted by other infiltrating cell types. Most importantly, these markers have not, in themselves, provided additional therapeutic pathways.

Therefore, there is a need to identify pathways associated with LUAD and LUSC in ex- and current smokers, which may have therapeutic potential.

Previous studies^37−40^ have concentrated on identifying tumor-specific transcriptomic or proteomic signatures in comparison to normal adjacent tissue, examining LUSC and/or LUAD separately. Often, the aim is to improve the diagnostic identification of tumors with respect to normal tissue rather than subtype-specific identification. The key findings from these studies have been that there are a number of gene and protein biomarkers of each tumor type in comparison with normal tissue controls, e.g., keratin expression raised in LUSC and biological pathway analysis identified cell adhesion and skin barrier pathways upregulated in LUSC and exocytosis, surfactant, and organic substance exposure upregulated in LUAD. The key challenge in these studies is that tumor tissue is more clonal and less complex than adjacent tissue, and thus, there is much overlap between the DEGs and DEPs of LUAD and LUSC compared to NAT separately due to commonality in the nature of tumor tissues in general. To address this, Stewart et al.^41^ performed a small-scale study comparing LUAD and LUSC directly and compared their data with reanalyzed data sets from Kikuchi et al. and Faruki et al. There was very little overlap in the DEPs and DEGs in these data sets, some of which were not designed for this comparison, and thus, there is a need for a direct comparison of these two tumor types, which would eliminate the commonality in these tissue types and might provide better additional useful knowledge in this area.

Here, we performed a full transcriptomic and global proteomic survey of both LUAD and LUSC in comparison with each other and also with NAT (proteomes) or with PBMC (transcriptomes), with the aim of identifying potential therapeutic pathways.

We found that, as expected, tissue transcriptomes or proteomes do not resemble those of either PBMC or NAT, respectively. Pleasingly, the NSCLC subtypes can also be differentiated from each other. We were easily able to separate the groups in each comparison by means of PCA of their gene counts and protein peptide intensities. At the gene level, the expression of the lncRNA transcript AFAP1-AS1 was a key component driving variation within LUAD. This transcript has been shown to predict poor prognosis for lung cancer in general.^42^ Keratin expression was the predominant feature of the separation between LUSC and LUAD. At the protein level, differences in expression of the known marker napsin-A^43^ drove the separation between LUSC and LUAD, along with several proinflammatory metal-binding proteins.

### DEGs between NSCLC and PBMCs

For both LUAD and LUSC, we observed many similar DEGs between tumors and PBMCs related to extracellular matrix remodeling and cell structure with DEGs of several collagen and keratin genes, such as COL1A1 and KRT19; cell adhesion, growth factors, and signaling, such as pulmonary surfactant-associated protein A2 (SFTPA2), EGFR, vascular endothelial growth factor receptor 2 (KDR), and proto-oncogene tyrosine-protein kinase ROS (ROS1); and cell differentiation, such as transcription factor SOX2, neurogenic locus notch homologue protein 3 (NOTCH3), and tumor protein 63 (TP63) (Tables S7 and S8). These findings are consistent with previous observations for NSCLC^33,44^ and were reflected in our functional analysis. Pulmonary surfactant SFTPA2 and its associated mutations were recently identified for use as a serum biomarker of NSCLC.^5^ This is suggestive of common processes in tumorigenesis and also potentially clonality in tumor tissue versus heterogeneous normal tissue. Additionally, we identified some of the same SFTPA2 mutations in several of our donors.^8^ Also, from the DEG expression patterns, LUAD was differentiated from LUSC, in addition to numerous protein biomarkers, by the expression of ENSG00000273132, which is an antisense transcript for receptor-related protein 11 (LRP11). This has been implicated in skin, thyroid, and breast cancer but not previously described in lung cancer.^45,46^

Metabolic processing genes, indoleamine 2,3-dioxygenase 1 (IDO1) and fatty acid synthase (FASN), were also both DEGs in both NSCLC subtypes relative to PBMCs and for which targeting drugs are either approved or in clinical trials.^47^

### DEGs between NSCLC Subtypes

We found two notable differences in gene expression in relation to immune inhibition between NSCLC subtypes as previously observed:^48^ fibrinogen-like protein 1 (FGL1) was a DEG in LUAD relative to LUSC. FGL1 has been identified as a T-cell suppressor through its action as a ligand of LAG-3.^49^ Autoimmune checkpoint gene V-set domain-containing T-cell activation (VTCN1)^50^ was a DEG in LUSC relative to LUAD. These observations for FGL1 in LUAD and VTCN1 in LUSC support their potential as subtype-specific targets for checkpoint inhibitor drugs (Tables S9 and S15).

### DEPs between NSCLC and NAT

Ribosomal proteins, such as small ribosomal subunit proteins eS19 and uS10 (RPS19, RPS20), were differentially expressed between both NSCLC subtypes and NAT. These and other ribosomal proteins are implicated in regulation of TP53^51^ and are indicative of the changes between tumor and normal tissue seen in the functional analysis identification of pathways related to protein translation and RNA-related processes (Figure S8, Tables S13, and S14).

### DEPs between NSCLC Subtypes

As expected, between NSCLC subtypes, napsin-A (NAPSA) was both a DEG and DEP in LUAD relative to LUSC, and thyroid transcription factor 1 (NKX2–1) was a DEG, supporting their known utility as immunohistological LUAD classifiers (Tables S9 and S15).^43^ A previously utilized machine learning NSCLC classification model of DEPs, in addition to NAPSA, identified the DEP anterior gradient protein 3 (AGR3) as a feature of LUAD and DEPs of KRT5 and SERPINB5 as features of LUSC, which were also present in our observations^48^ (Table S15).

Other proteins in LUSC differentially expressed in comparison to LUAD included poly ADP-ribose polymerase 1 (PARP1), a target for the DNA repair inhibitor Olaparib. Results for PARP1 inhibition in a recent NSCLC trial for patients with homologous repair deficiency were inconclusive;^52^ however, another trial is ongoing (NCT03976362). Epigenome histone deacetylases (HDAC1, HDAC2) were also DEPs for LUSC and are under trial as a target for entinostat as an inhibitor/chemosensitizer (NCT05053971).^53,54^ DEP RAC-alpha serine/threonine-protein kinase (AKT1) has been identified, playing a role in transdifferentiation of LUAD to LUSC.^55^ DEPs, transferrin receptor protein 1 (TFRC) and phosphoserine aminotransferase (PSAT1), have been previously identified as characteristic of a LUSC subtype related to changes in metabolic signaling and oxidative stress.^56^

For LUAD, DEPs compared to LUSC included mucin-1 (MUC1), a target for salinomycin,^57^ and serine/threonine-protein kinase mTOR (MTOR), which has been identified as a chemosensitizing target,^58^ while enzymes transglutaminase 2 (TGM2) and sterol O-acyltransferase 1 (SOAT1) have been identified as targets for inhibition^59,60^ (Table S15). An intriguing LUAD DEP is B-cell lymphoma/leukemia 10 (BCL10), which has a role in inflammation as part of the multiprotein complex in the NF-κB pathway^61,62^ and therefore may relate to the activation of EGFR.^63^ In B-cell lymphomas, trials for inhibitors targeting another protein in the same complex, mucosa-associated lymphoid tissue protein 1 (MALT1), are ongoing, as well as efforts to understand their efficacy in solid cancers.^64^

The high degree of enrichment of both DEGs and DEPs in LUSC compared to LUAD in our study is reflected to some degree in previous studies^37−41^ and may reflect a generalizable increased difference from precursor cells in LUSC due to the number of increased gene transcripts required for squamous differentiation. Shared transcriptome pathways of both subtypes compared to PBMCs suggest that there are common pathways in tumor versus nontumor cells related to ECM remodeling and immune system activation, also seen in previous studies. Our data suggest that GO pathways specific to LUSC at the gene level and also at the protein level are generally related to cell ECM deposition, epidermal differentiation, and increased cell/cell contacts, whereas those specific to LUAD are fewer and mostly related to sugar biosynthesis/metabolism.

Surprisingly, the reactome pathway analysis for LUSC identifies only the formation of the cornified envelope at both the gene and protein level, although this is a well-known pathway in squamous metaplasia.^65^ LUAD reactome pathways include Dectin, as well as glycan biosynthesis or metabolic pathway participants, such as GALT1C1 and GALNT. Dectin has recently been identified as a prognostic marker in LUAD,^66^ with some types associated with checkpoint inhibitor expression. GALNT-3 has recently been implicated in lung cancer development and regulation of the tumor microenvironment using in vitro and in vivo models.^67^

Previously, Stewart et al.^41^ identified nine DEPs shared across three compared studies: 7 were DEPs in LUSC and 2 in LUAD. Of these, we also identified 3 of the LUSC DEPs (PKP1, monocarboxylate transporter 1 (SLC16A1), and solute carrier family 2, facilitated glucose transporter member 1 (SLC2A1)). The four keratins and collagen they identified as DEPs in LUSC were not identified across all samples and therefore were not quantified in our data. LIM domain only protein 7 (LMO7) was also a DEP in LUAD in our data but at a log_2_FC of 0.83 and an adjusted *p*-value of 0.42. The other LUAD DEP they identified, ATP-binding cassette subfamily F member 3 (ABCF3), was almost equally expressed for both NSCLC subtypes in our data (Table S15). Differences between our data and these findings likely also reflect differences in study design and proteomic approach combined with relatively small patient cohorts. Our study was specifically designed to perform the LUSC and LUAD comparison, and the discovery of well-known markers of difference in our DEG and DEP data sets provides some confirmation of the validity of the findings.

The main limitations in our design were that we were unable to compare NSCLC transcriptomes to NAT transcriptomes and that we examined a modestly sized cohort. Use of PBMCs as control cells for DEG analysis will have confounded our observations to some extent, particularly with respect to the expression of keratins and other genes that phenotypically differentiate lung tissues from blood. Hence, this limits the interpretation of the NSCLC and PBMC DEG comparison but not the NSCLC subtype DEG comparison.

In conclusion, our study confirms previous findings of significant differences in both gene expression and protein expression patterns in both LUSC and LUAD compared to those of control tissue samples. Furthermore, differences in expression between these two tumor types have revealed the most significant differences in pathways between these two tumor types and uncovered novel coding and noncoding gene and protein expression patterns, which will prove useful in the characterization and therapeutic development for treatment of these two tumor types.

## Data Availability

RNaseq data have been deposited at the European Genome-phenome Archive (EGA) under EGA Study ID: EGAS00001005499. The mass spectrometry proteomics data have been deposited to the ProteomeXchange Consortium via the PRIDE^68^ partner repository with the data set identifiers PXD054390 and https://doi.org/10.6019/PXD054390. Supporting Information is available on Github: https://github.com/ab604/lung-global-supplement and Zenodo DOI: https://doi.org/10.5281/zenodo.13327662.

## Supporting Information Available

PCA biplots of NSCLC subtypes PBMC and NAT comparisons (Figures S1 and S2); volcano plots of NSCLC subtypes PBMC and NAT comparisons (Figures S3 and S4); heatmaps of NSCLC DEGs and DEPs (Figures S5–S7); and bar plots of functional enrichment between NSCLC subtypes and PBMCs and NAT (Figure S8) (PDF)
Gene counts from the HISAT2 alignments estimated by featureCounts (Tables S1–S3); gene counts from transcript classification by Salmon (Tables S4–S6); differential gene expression edgeR outputs (Tables S7–S9); peaks normalized top 3 peptide intensities (Tables S10–S12); differential protein expression DEqMS outputs (Tables S13–S15); and functional enrichment analysis g:Profiler outputs (Tables S16 and S17) (ZIP)

## References

[ref1] Types of Lung Cancer. https://www.cancerresearchuk.org/about-cancer/lung-cancer/stages-types-grades/types.

[ref2] Cancer Survival in England, Cancers Diagnosed 2016 to 2020, Followed up to 2021. https://digital.nhs.uk/data-and-information/publications/statistical/cancer-survival-in-england/cancers-diagnosed-2016-to-2020-followed-up-to-2021.

[ref3] Cancer Survival in England - Office for National Statistics. https://www.ons.gov.uk/peoplepopulationandcommunity/healthandsocialcare/conditionsanddiseases/bulletins/cancersurvivalinengland/stageatdiagnosisandchildhoodpatientsfollowedupto2018.

[ref4] Wang W.; Liu H.; Li G. What’s the Difference Between Lung Adenocarcinoma and Lung Squamous Cell Carcinoma? Evidence from a Retrospective Analysis in a Cohort of Chinese Patients. Front. Endocrinol. 2022, 13, 947443 10.3389/fendo.2022.947443. PMC9465444 36105402

[ref5] Papier K.; Atkins J. R.; Tong T. Y. N.; Gaitskell K.; Desai T.; Ogamba C. F.; Parsaeian M.; Reeves G. K.; Mills I. G.; Key T. J.; Smith-Byrne K.; Travis R. C. Identifying Proteomic Risk Factors for Cancer Using Prospective and Exome Analyses of 1463 Circulating Proteins and Risk of 19 Cancers in the UK Biobank. Nat. Commun. 2024, 15 (1), 4010 10.1038/s41467-024-48017-6. 38750076 PMC11096312

[ref6] Asakura K.; Kadota T.; Matsuzaki J.; Yoshida Y.; Yamamoto Y.; Nakagawa K.; Takizawa S.; Aoki Y.; Nakamura E.; Miura J.; Sakamoto H.; Kato K.; Watanabe S.; Ochiya T. A miRNA-Based Diagnostic Model Predicts Resectable Lung Cancer in Humans with High Accuracy. Commun. Biol. 2020, 3 (1), 134 10.1038/s42003-020-0863-y. 32193503 PMC7081195

[ref7] Jamal-Hanjani M.; Wilson G. A.; McGranahan N.; Birkbak N. J.; Watkins T. B. K.; Veeriah S.; Shafi S.; Johnson D. H.; Mitter R.; Rosenthal R.; Salm M.; Horswell S.; Escudero M.; Matthews N.; Rowan A.; Chambers T.; Moore D. A.; Turajlic S.; Xu H.; Lee S.-M.; Forster M. D.; Ahmad T.; Hiley C. T.; Abbosh C.; Falzon M.; Borg E.; Marafioti T.; Lawrence D.; Hayward M.; Kolvekar S.; Panagiotopoulos N.; Janes S. M.; Thakrar R.; Ahmed A.; Blackhall F.; Summers Y.; Shah R.; Joseph L.; Quinn A. M.; Crosbie P. A.; Naidu B.; Middleton G.; Langman G.; Trotter S.; Nicolson M.; Remmen H.; Kerr K.; Chetty M.; Gomersall L.; Fennell D. A.; Nakas A.; Rathinam S.; Anand G.; Khan S.; Russell P.; Ezhil V.; Ismail B.; Irvin-Sellers M.; Prakash V.; Lester J. F.; Kornaszewska M.; Attanoos R.; Adams H.; Davies H.; Dentro S.; Taniere P.; O’Sullivan B.; Lowe H. L.; Hartley J. A.; Iles N.; Bell H.; Ngai Y.; Shaw J. A.; Herrero J.; Szallasi Z.; Schwarz R. F.; Stewart A.; Quezada S. A.; Le Quesne J.; Van Loo P.; Dive C.; Hackshaw A.; Swanton C. Tracking the Evolution of NonSmall-Cell Lung Cancer. New England Journal of Medicine 2017, 376 (22), 2109–2121. 10.1056/NEJMoa1616288. 28445112

[ref8] Nicholas B.; Bailey A.; McCann K. J.; Wood O.; Currall E.; Johnson P.; Elliott T.; Ottensmeier C.; Skipp P. Proteogenomics Guided Identification of Functional Neoantigens in Non-Small Cell Lung Cancer. bioRxiv 2024, 10.1101/2024.05.30.596609. PMC13601597 42270771

[ref9] Chen S.; Zhou Y.; Chen Y.; Gu J. Fastp: An Ultra-Fast All-in-One FASTQ Preprocessor. Bioinformatics 2018, 34 (17), i884–i890. 10.1093/bioinformatics/bty560. 30423086 PMC6129281

[ref10] Kim D.; Paggi J. M.; Park C.; Bennett C.; Salzberg S. L. Graph-Based Genome Alignment and Genotyping with HISAT2 and HISAT-Genotype. Nat. Biotechnol. 2019, 37 (8), 907–915. 10.1038/s41587-019-0201-4. 31375807 PMC7605509

[ref11] Liao Y.; Smyth G. K.; Shi W. featureCounts: An Efficient General Purpose Program for Assigning Sequence Reads to Genomic Features. Bioinformatics 2014, 30 (7), 923–930. 10.1093/bioinformatics/btt656. 24227677

[ref12] Srivastava A.; Malik L.; Sarkar H.; Patro R. A Bayesian Framework for Inter-Cellular Information Sharing Improves dscRNA-Seq Quantification. Bioinformatics 2020, 36 (Suppl_1), i292–i299. 10.1093/bioinformatics/btaa450. 32657394 PMC7355277

[ref13] Chen Y.; Lun A. T. L.; McCarthy D.; Chen L.; Baldoni P.; Ritchie M. E.; Phipson B.; Hu Y.; Zhou X.; Robinson M. D.; Smyth G. K. edgeR, 2017. https://doi.org/10.18129/B9.BIOC.EDGER.

[ref14] Love M. I.; Huber W.; Anders S. Moderated Estimation of Fold Change and Dispersion for RNA-Seq Data with DESeq2. Genome Biol. 2014, 15, 550 10.1186/s13059-014-0550-8. 25516281 PMC4302049

[ref15] Blighe K.; Brown A.-L.; Carey V.; Hooiveld G.; Lun A. PCAtools, 2019. https://doi.org/10.18129/B9.BIOC.PCATOOLS.

[ref16] Blighe K. EnhancedVolcano, 2018. https://doi.org/10.18129/B9.BIOC.ENHANCEDVOLCANO.

[ref17] Pheatmap: Pretty Heatmaps, 2010. https://doi.org/10.32614/cran.package.pheatmap.

[ref18] Wickham H. Ggplot2: Elegant Graphics for Data Analysis; Springer, 2016.

[ref19] Bligh E. G.; Dyer W. J. A RAPID METHOD OF TOTAL LIPID EXTRACTION AND PURIFICATION. Canadian Journal of Biochemistry and Physiology 1959, 37 (8), 911–917. 10.1139/o59-099. 13671378

[ref20] Zhang J.; Xin L.; Shan B.; Chen W.; Xie M.; Yuen D.; Zhang W.; Zhang Z.; Lajoie G. A.; Ma B. PEAKS DB: De Novo Sequencing Assisted Database Search for Sensitive and Accurate Peptide Identification. Mol. Cell. Proteomics 2012, 11 (4), M111.010587 10.1074/mcp.M111.010587. PMC3322562 22186715

[ref21] Tran N. H.; Zhang X.; Xin L.; Shan B.; Li M. De Novo Peptide Sequencing by Deep Learning. Proc. Natl. Acad. Sci. U.S.A. 2017, 114, 8247 10.1073/pnas.1705691114. 28720701 PMC5547637

[ref22] Lin H.; He L.; Ma B. A Combinatorial Approach to the Peptide Feature Matching Problem for Label-Free Quantification. Bioinformatics 2013, 29 (14), 1768–1775. 10.1093/bioinformatics/btt274. 23665772

[ref23] DEqMS. http://bioconductor.org/packages/DEqMS/.

[ref24] Kolberg L.; Raudvere U.; Kuzmin I.; Vilo J.; Peterson H. Gprofiler2–an R Package for Gene List Functional Enrichment Analysis and Namespace Conversion Toolset g:Profiler. F1000Res. 2020, 9, 709 10.12688/f1000research.24956.2. PMC7859841 33564394

[ref25] Van Loo P.; Nordgard S. H.; Lingjærde O. C.; Russnes H. G.; Rye I. H.; Sun W.; Weigman V. J.; Marynen P.; Zetterberg A.; Naume B.; Perou C. M.; Bo̷rresen-Dale A.-L.; Kristensen V. N. Allele-Specific Copy Number Analysis of Tumors. Proc. Natl. Acad. Sci. U. S. A. 2010, 107 (39), 16910–16915. 10.1073/pnas.1009843107. 20837533 PMC2947907

[ref26] Raine K. M.; Van Loo P.; Wedge D. C.; Jones D.; Menzies A.; Butler A. P.; Teague J. W.; Tarpey P.; Nik-Zainal S.; Campbell P. J. ascatNgs: Identifying Somatically Acquired Copy-Number Alterations from Whole-Genome Sequencing Data. Curr. Protoc. Bioinformatics 2016, 56 (1), 15.9.1–15.9.17. 10.1002/cpbi.17. PMC6097604 27930809

[ref27] Peng H.; Wang H.; Kong W.; Li J.; Goh W. W. B. Optimizing Differential Expression Analysis for Proteomics Data via High-Performing Rules and Ensemble Inference. Nat. Commun. 2024, 15 (1), 3922 10.1038/s41467-024-47899-w. 38724498 PMC11082229

[ref28] Pertea M.; Kim D.; Pertea G. M.; Leek J. T.; Salzberg S. L. Transcript-Level Expression Analysis of RNA-Seq Experiments with HISAT. StringTie and Ballgown. Nature Protocols 2016, 11 (9), 1650–1667. 10.1038/nprot.2016.095. 27560171 PMC5032908

[ref29] Ashburner M.; Ball C. A.; Blake J. A.; Botstein D.; Butler H.; Cherry J. M.; Davis A. P.; Dolinski K.; Dwight S. S.; Eppig J. T.; Harris M. A.; Hill D. P.; Issel-Tarver L.; Kasarskis A.; Lewis S.; Matese J. C.; Richardson J. E.; Ringwald M.; Rubin G. M.; Sherlock G. Gene Ontology: Tool for the Unification of Biology. Nat. Genet. 2000, 25 (1), 25–29. 10.1038/75556. 10802651 PMC3037419

[ref30] Aleksander S. A.; Balhoff J.; Carbon S.; Cherry J. M.; Drabkin H. J.; Ebert D.; Feuermann M.; Gaudet P.; Harris N. L.; Hill D. P.; Lee R.; Mi H.; Moxon S.; Mungall C. J.; Muruganugan A.; Mushayahama T.; Sternberg P. W.; Thomas P. D.; Van Auken K.; Ramsey J.; Siegele D. A.; Chisholm R. L.; Fey P.; Aspromonte M. C.; Nugnes M. V.; Quaglia F.; Tosatto S.; Giglio M.; Nadendla S.; Antonazzo G.; Attrill H.; dos Santos G.; Marygold S.; Strelets V.; Tabone C. J.; Thurmond J.; Zhou P.; Ahmed S. H.; Asanitthong P.; Luna Buitrago D.; Erdol M. N.; Gage M. C.; Ali Kadhum M.; Li K. Y. C.; Long M.; Michalak A.; Pesala A.; Pritazahra A.; Saverimuttu S. C. C.; Su R.; Thurlow K. E.; Lovering R. C.; Logie C.; Oliferenko S.; Blake J.; Christie K.; Corbani L.; Dolan M. E.; Drabkin H. J.; Hill D. P.; Ni L.; Sitnikov D.; Smith C.; Cuzick A.; Seager J.; Cooper L.; Elser J.; Jaiswal P.; Gupta P.; Jaiswal P.; Naithani S.; Lera-Ramirez M.; Rutherford K.; Wood V.; De Pons J. L.; Dwinell M. R.; Hayman G. T.; Kaldunski M. L.; Kwitek A. E.; Laulederkind S. J. F.; Tutaj M. A.; Vedi M.; Wang S.-J.; D’Eustachio P.; Aimo L.; Axelsen K.; Bridge A.; Hyka-Nouspikel N.; Morgat A.; Aleksander S. A.; Cherry J. M.; Engel S. R.; Karra K.; Miyasato S. R.; Nash R. S.; Skrzypek M. S.; Weng S.; Wong E. D.; Bakker E.; Berardini T. Z.; Reiser L.; Auchincloss A.; Axelsen K.; Argoud-Puy G.; Blatter M.-C.; Boutet E.; Breuza L.; Bridge A.; Casals-Casas C.; Coudert E.; Estreicher A.; Livia Famiglietti M.; Feuermann M.; Gos A.; Gruaz-Gumowski N.; Hulo C.; Hyka-Nouspikel N.; Jungo F.; Le Mercier P.; Lieberherr D.; Masson P.; Morgat A.; Pedruzzi I.; Pourcel L.; Poux S.; Rivoire C.; Sundaram S.; Bateman A.; Bowler-Barnett E.; Bye-A-Jee H.; Denny P.; Ignatchenko A.; Ishtiaq R.; Lock A.; Lussi Y.; Magrane M.; Martin M. J.; Orchard S.; Raposo P.; Speretta E.; Tyagi N.; Warner K.; Zaru R.; Diehl A. D.; Lee R.; Chan J.; Diamantakis S.; Raciti D.; Zarowiecki M.; Fisher M.; James-Zorn C.; Ponferrada V.; Zorn A.; Ramachandran S.; Ruzicka L.; Westerfield M.; Aleksander S. A.; Balhoff J.; Carbon S.; Cherry J. M.; Drabkin H. J.; Ebert D.; Feuermann M.; Gaudet P.; Harris N. L.; Hill D. P.; Lee R.; Mi H.; Moxon S.; Mungall C. J.; Muruganugan A.; Mushayahama T.; Sternberg P. W.; Thomas P. D.; Van Auken K.; Ramsey J.; Siegele D. A.; Chisholm R. L.; Fey P.; Aspromonte M. C.; Nugnes M. V.; Quaglia F.; Tosatto S.; Giglio M.; Nadendla S.; Antonazzo G.; Attrill H.; dos Santos G.; Marygold S.; Strelets V.; Tabone C. J.; Thurmond J.; Zhou P.; Ahmed S. H.; Asanitthong P.; Luna Buitrago D.; Erdol M. N.; Gage M. C.; Ali Kadhum M.; Li K. Y. C.; Long M.; Michalak A.; Pesala A.; Pritazahra A.; Saverimuttu S. C. C.; Su R.; Thurlow K. E.; Lovering R. C.; Logie C.; Oliferenko S.; Blake J.; Christie K.; Corbani L.; Dolan M. E.; Drabkin H. J.; Hill D. P.; Ni L.; Sitnikov D.; Smith C.; Cuzick A.; Seager J.; Cooper L.; Elser J.; Jaiswal P.; Gupta P.; Jaiswal P.; Naithani S.; Lera-Ramirez M.; Rutherford K.; Wood V.; De Pons J. L.; Dwinell M. R.; Hayman G. T.; Kaldunski M. L.; Kwitek A. E.; Laulederkind S. J. F.; Tutaj M. A.; Vedi M.; Wang S.-J.; D’Eustachio P.; Aimo L.; Axelsen K.; Bridge A.; Hyka-Nouspikel N.; Morgat A.; Aleksander S. A.; Cherry J. M.; Engel S. R.; Karra K.; Miyasato S. R.; Nash R. S.; Skrzypek M. S.; Weng S.; Wong E. D.; Bakker E.; Berardini T. Z.; Reiser L.; Auchincloss A.; Axelsen K.; Argoud-Puy G.; Blatter M.-C.; Boutet E.; Breuza L.; Bridge A.; Casals-Casas C.; Coudert E.; Estreicher A.; Livia Famiglietti M.; Feuermann M.; Gos A.; Gruaz-Gumowski N.; Hulo C.; Hyka-Nouspikel N.; Jungo F.; Le Mercier P.; Lieberherr D.; Masson P.; Morgat A.; Pedruzzi I.; Pourcel L.; Poux S.; Rivoire C.; Sundaram S.; Bateman A.; Bowler-Barnett E.; Bye-A-Jee H.; Denny P.; Ignatchenko A.; Ishtiaq R.; Lock A.; Lussi Y.; Magrane M.; Martin M. J.; Orchard S.; Raposo P.; Speretta E.; Tyagi N.; Warner K.; Zaru R.; Diehl A. D.; Lee R.; Chan J.; Diamantakis S.; Raciti D.; Zarowiecki M.; Fisher M.; James-Zorn C.; Ponferrada V.; Zorn A.; Ramachandran S.; Ruzicka L.; Westerfield M. The Gene Ontology Knowledgebase in 2023. Genetics 2023, 224 (1), iyad031 10.1093/genetics/iyad031. 36866529 PMC10158837

[ref31] Milacic M.; Beavers D.; Conley P.; Gong C.; Gillespie M.; Griss J.; Haw R.; Jassal B.; Matthews L.; May B.; Petryszak R.; Ragueneau E.; Rothfels K.; Sevilla C.; Shamovsky V.; Stephan R.; Tiwari K.; Varusai T.; Weiser J.; Wright A.; Wu G.; Stein L.; Hermjakob H.; D’Eustachio P. The Reactome Pathway Knowledgebase 2024. Nucleic Acids Res. 2024, 52 (D1), D672–D678. 10.1093/nar/gkad1025. 37941124 PMC10767911

[ref32] Hanahan D. Hallmarks of Cancer: New Dimensions. Cancer Discovery 2022, 12 (1), 31–46. 10.1158/2159-8290.CD-21-1059. 35022204

[ref33] Sánchez-Danés A.; Blanpain C. Deciphering the Cells of Origin of Squamous Cell Carcinomas. Nature Reviews Cancer 2018, 18 (9), 549–561. 10.1038/s41568-018-0024-5. 29849070 PMC7170720

[ref34] Mok T.; Camidge D. R.; Gadgeel S. M.; Rosell R.; Dziadziuszko R.; Kim D.-W.; Pérol M.; Ou S.-H. I.; Ahn J. S.; Shaw A. T.; Bordogna W.; Smoljanović V.; Hilton M.; Ruf T.; Noé J.; Peters S. Updated Overall Survival and Final Progression-Free Survival Data for Patients with Treatment-Naive Advanced ALK-Positive Non-Small-Cell Lung Cancer in the ALEX Study. Annals of Oncology 2020, 31 (8), 1056–1064. 10.1016/j.annonc.2020.04.478. 32418886

[ref35] Ramalingam S. S.; Vansteenkiste J.; Planchard D.; Cho B. C.; Gray J. E.; Ohe Y.; Zhou C.; Reungwetwattana T.; Cheng Y.; Chewaskulyong B.; Shah R.; Cobo M.; Lee K. H.; Cheema P.; Tiseo M.; John T.; Lin M.-C.; Imamura F.; Kurata T.; Todd A.; Hodge R.; Saggese M.; Rukazenkov Y.; Soria J.-C. Overall Survival with Osimertinib in Untreated, *EGFR*-Mutated Advanced NSCLC. New England Journal of Medicine 2020, 382 (1), 41–50. 10.1056/NEJMoa1913662. 31751012

[ref36] Cooper A. J.; Sequist L. V.; Lin J. J. Third-Generation EGFR and ALK Inhibitors: Mechanisms of Resistance and Management. Nature Reviews Clinical Oncology 2022, 19 (8), 499–514. 10.1038/s41571-022-00639-9. PMC9621058 35534623

[ref37] Kikuchi T.; Hassanein M.; Amann J. M.; Liu Q.; Slebos R. J. C.; Rahman S. M. J.; Kaufman J. M.; Zhang X.; Hoeksema M. D.; Harris B. K.; Li M.; Shyr Y.; Gonzalez A. L.; Zimmerman L. J.; Liebler D. C.; Massion P. P.; Carbone D. P. In-Depth Proteomic Analysis of Nonsmall Cell Lung Cancer to Discover Molecular Targets and Candidate Biomarkers. Molecular & Cellular Proteomics 2012, 11 (10), 916–932. 10.1074/mcp.M111.015370. 22761400 PMC3494148

[ref38] Li L.; Wei Y.; To C.; Zhu C.-Q.; Tong J.; Pham N.-A.; Taylor P.; Ignatchenko V.; Ignatchenko A.; Zhang W.; Wang D.; Yanagawa N.; Li M.; Pintilie M.; Liu G.; Muthuswamy L.; Shepherd F. A.; Tsao M. S.; Kislinger T.; Moran M. F. Integrated Omic Analysis of Lung Cancer Reveals Metabolism Proteome Signatures with Prognostic Impact. Nat. Commun. 2014, 5 (1), 5469 10.1038/ncomms6469. 25429762

[ref39] Faruki H.; Mayhew G. M.; Serody J. S.; Hayes D. N.; Perou C. M.; Lai-Goldman M. Lung Adenocarcinoma and Squamous Cell Carcinoma Gene Expression Subtypes Demonstrate Significant Differences in Tumor Immune Landscape. Journal of Thoracic Oncology 2017, 12 (6), 943–953. 10.1016/j.jtho.2017.03.010. 28341226 PMC6557266

[ref40] Chen J. W.; Dhahbi J. Lung Adenocarcinoma and Lung Squamous Cell Carcinoma Cancer Classification, Biomarker Identification, and Gene Expression Analysis Using Overlapping Feature Selection Methods. Sci. Rep. 2021, 11 (1), 13323 10.1038/s41598-021-92725-8. 34172784 PMC8233431

[ref41] Stewart P. A.; Parapatics K.; Welsh E. A.; Müller A. C.; Cao H.; Fang B.; Koomen J. M.; Eschrich S. A.; Bennett K. L.; Haura E. B. A Pilot Proteogenomic Study with Data Integration Identifies MCT1 and GLUT1 as Prognostic Markers in Lung Adenocarcinoma. PLoS One 2015, 10 (11), e0142162 10.1371/journal.pone.0142162. 26539827 PMC4634858

[ref42] Zhong Y.; Yang L.; Xiong F.; He Y.; Tang Y.; Shi L.; Fan S.; Li Z.; Zhang S.; Gong Z.; Guo C.; Liao Q.; Zhou Y.; Zhou M.; Xiang B.; Li X.; Li Y.; Zeng Z.; Li G.; Xiong W. Long Non-Coding RNA AFAP1-AS1 Accelerates Lung Cancer Cells Migration and Invasion by Interacting with SNIP1 to Upregulate c-Myc. Signal Transduction Targeted Ther. 2021, 6 (1), 240 10.1038/s41392-021-00562-y. PMC8225811 34168109

[ref43] Ao M.-H.; Zhang H.; Sakowski L.; Sharma R.; Illei P. B.; Gabrielson E.; Askin F.; Li Q. K. The Utility of a Novel Triple Marker (Combination of TTF1, Napsin A, and P40) in the Subclassification of Nonsmall Cell Lung Cancer. Human Pathology 2014, 45 (5), 926–934. 10.1016/j.humpath.2014.01.005. 24746197 PMC4178947

[ref44] Uhlen M.; Zhang C.; Lee S.; Sjöstedt E.; Fagerberg L.; Bidkhori G.; Benfeitas R.; Arif M.; Liu Z.; Edfors F.; Sanli K.; von Feilitzen K.; Oksvold P.; Lundberg E.; Hober S.; Nilsson P.; Mattsson J.; Schwenk J. M.; Brunnström H.; Glimelius B.; Sjöblom T.; Edqvist P.-H.; Djureinovic D.; Micke P.; Lindskog C.; Mardinoglu A.; Ponten F. A Pathology Atlas of the Human Cancer Transcriptome. Science 2017, 357 (6352), eaan2507 10.1126/science.aan2507. 28818916

[ref45] Goedert L.; Plaça J. R.; Fuziwara C. S.; Machado M. C. R.; Plaça D. R.; Almeida P. P.; Sanches T. P.; dos Santos J. F.; Corveloni A. C.; Pereira I. E. G.; de Castro M. M.; Kimura E. T.; Silva W. A.; Espreafico E. M. Identification of Long Noncoding RNAs Deregulated in Papillary Thyroid Cancer and Correlated with BRAFV600E Mutation by Bioinformatics Integrative Analysis. Sci. Rep. 2017, 7 (1), 1662 10.1038/s41598-017-01957-0. 28490781 PMC5431778

[ref46] Li P.; Zeng Y.; Chen Y.; Huang P.; Chen X.; Zheng W. LRP11-AS1 Promotes the Proliferation and Migration of Triple Negative Breast Cancer Cells via the miR-149–3p/NRP2 Axis. Cancer Cell Int. 2022, 22 (1), 116 10.1186/s12935-022-02536-8. 35279146 PMC8917722

[ref47] Stine Z. E.; Schug Z. T.; Salvino J. M.; Dang C. V. Targeting Cancer Metabolism in the Era of Precision Oncology. Nat. Rev. Drug Discovery 2022, 21 (2), 141–162. 10.1038/s41573-021-00339-6. 34862480 PMC8641543

[ref48] Lehtiö J.; Arslan T.; Siavelis I.; Pan Y.; Socciarelli F.; Berkovska O.; Umer H. M.; Mermelekas G.; Pirmoradian M.; Jönsson M.; Brunnström H.; Brustugun O. T.; Purohit K. P.; Cunningham R.; Foroughi Asl H.; Isaksson S.; Arbajian E.; Aine M.; Karlsson A.; Kotevska M.; Gram Hansen C.; Drageset Haakensen V.; Helland Å.; Tamborero D.; Johansson H. J.; Branca R. M.; Planck M.; Staaf J.; Orre L. M. Proteogenomics of Non-Small Cell Lung Cancer Reveals Molecular Subtypes Associated with Specific Therapeutic Targets and Immune-Evasion Mechanisms. Nature Cancer 2021, 2 (11), 1224–1242. 10.1038/s43018-021-00259-9. 34870237 PMC7612062

[ref49] Wang J.; Sanmamed M. F.; Datar I.; Su T. T.; Ji L.; Sun J.; Chen L.; Chen Y.; Zhu G.; Yin W.; Zheng L.; Zhou T.; Badri T.; Yao S.; Zhu S.; Boto A.; Sznol M.; Melero I.; Vignali D. A. A.; Schalper K.; Chen L. Fibrinogen-Like Protein 1 Is a Major Immune Inhibitory Ligand of LAG-3. Cell 2019, 176 (1–2), 334–347.e12. 10.1016/j.cell.2018.11.010. 30580966 PMC6365968

[ref50] Wei J.; Loke P.; Zang X.; Allison J. P. Tissue-Specific Expression of B7x Protects from CD4 T Cellmediated Autoimmunity. Journal of Experimental Medicine 2011, 208 (8), 1683–1694. 10.1084/jem.20100639. 21727190 PMC3149222

[ref51] Kang J.; Brajanovski N.; Chan K. T.; Xuan J.; Pearson R. B.; Sanij E. Ribosomal Proteins and Human Diseases: Molecular Mechanisms and Targeted Therapy. Signal Transduction and Targeted Therapy 2021, 6 (1), 323 10.1038/s41392-021-00728-8. 34462428 PMC8405630

[ref52] Fennell D. A.; Porter C.; Lester J.; Danson S.; Blackhall F.; Nicolson M.; Nixon L.; Gardner G.; White A.; Griffiths G.; Casbard A. Olaparib Maintenance Versus Placebo Monotherapy in Patients with Advanced Non-Small Cell Lung Cancer (PIN): A Multicentre, Randomised, Controlled, Phase 2 Trial. eClinicalMedicine 2022, 52, 101595 10.1016/j.eclinm.2022.101595. 35990583 PMC9386392

[ref53] Lau S. C. M.; Pan Y.; Velcheti V.; Wong K. K. Squamous Cell Lung Cancer: Current Landscape and Future Therapeutic Options. Cancer Cell 2022, 40 (11), 1279–1293. 10.1016/j.ccell.2022.09.018. 36270277

[ref54] Solta A.; Boettiger K.; Kovács I.; Lang C.; Megyesfalvi Z.; Ferk F.; Mišík M.; Hoetzenecker K.; Aigner C.; Kowol C. R.; Knasmueller S.; Grusch M.; Szeitz B.; Rezeli M.; Dome B.; Schelch K. Entinostat Enhances the Efficacy of Chemotherapy in Small Cell Lung Cancer Through S-Phase Arrest and Decreased Base Excision Repair. Clin. Cancer Res. 2023, 29 (22), 4644–4659. 10.1158/1078-0432.CCR-23-1795. 37725585 PMC10644001

[ref55] Quintanal-Villalonga A.; Taniguchi H.; Zhan Y. A.; Hasan M. M.; Chavan S. S.; Meng F.; Uddin F.; Allaj V.; Manoj P.; Shah N. S.; Chan J. M.; Ciampricotti M.; Chow A.; Offin M.; Ray-Kirton J.; Egger J. D.; Bhanot U. K.; Linkov I.; Asher M.; Roehrl M. H.; Ventura K.; Qiu J.; de Stanchina E.; Chang J. C.; Rekhtman N.; Houck-Loomis B.; Koche R. P.; Yu H. A.; Sen T.; Rudin C. M. Comprehensive Molecular Characterization of Lung Tumors Implicates AKT and MYC Signaling in Adenocarcinoma to Squamous Cell Transdifferentiation. J. Hematol. Oncol. 2021, 14 (1), 170 10.1186/s13045-021-01186-z. 34656143 PMC8520275

[ref56] Stewart P. A.; Welsh E. A.; Slebos R. J. C.; Fang B.; Izumi V.; Chambers M.; Zhang G.; Cen L.; Pettersson F.; Zhang Y.; Chen Z.; Cheng C.-H.; Thapa R.; Thompson Z.; Fellows K. M.; Francis J. M.; Saller J. J.; Mesa T.; Zhang C.; Yoder S.; DeNicola G. M.; Beg A. A.; Boyle T. A.; Teer J. K.; Ann Chen Y.; Koomen J. M.; Eschrich S. A.; Haura E. B. Proteogenomic Landscape of Squamous Cell Lung Cancer. Nat. Commun. 2019, 10 (1), 3578 10.1038/s41467-019-11452-x. 31395880 PMC6687710

[ref57] Daimon T.; Bhattacharya A.; Wang K.; Haratake N.; Nakashoji A.; Ozawa H.; Morimoto Y.; Yamashita N.; Kosaka T.; Oya M.; Kufe D. W. MUC1-C Is a Target of Salinomycin in Inducing Ferroptosis of Cancer Stem Cells. Cell Death Discov. 2024, 10 (1), 9 10.1038/s41420-023-01772-9. 38182558 PMC10770371

[ref58] Liu Y.; Azizian N. G.; Sullivan D. K.; Li Y. mTOR Inhibition Attenuates Chemosensitivity Through the Induction of Chemotherapy Resistant Persisters. Nat. Commun. 2022, 13 (1), 7047 10.1038/s41467-022-34890-6. 36396656 PMC9671908

[ref59] Wang Z.; Wang M.; Zhang M.; Xu K.; Zhang X.; Xie Y.; Zhang Y.; Chang C.; Li X.; Sun A.; He F. High-Affinity SOAT1 Ligands Remodeled Cholesterol Metabolism Program to Inhibit Tumor Growth. BMC Med. 2022, 20 (1), 292 10.1186/s12916-022-02436-8. 35941608 PMC9361549

[ref60] Malkomes P.; Lunger I.; Oppermann E.; Lorenz J.; Faqar-Uz-Zaman S. F.; Han J.; Bothur S.; Ziegler P.; Bankov K.; Wild P.; Bechstein W. O.; Rieger M. A. Transglutaminase 2 Is Associated with Adverse Colorectal Cancer Survival and Represents a Therapeutic Target. Cancer Gene Ther. 2023, 30 (10), 1346–1354. 10.1038/s41417-023-00641-y. 37443286 PMC10581896

[ref61] Rosebeck S.; Rehman A. O.; Lucas P. C.; McAllister-Lucas L. M. From MALT Lymphoma to the CBM Signalosome. Cell Cycle 2011, 10 (15), 2485–2496. 10.4161/cc.10.15.16923. 21750409 PMC3180188

[ref62] Taniguchi K.; Karin M. NF-κB, Inflammation, Immunity and Cancer: Coming of Age. Nature Reviews Immunology 2018, 18 (5), 309–324. 10.1038/nri.2017.142. 29379212

[ref63] Bivona T. G.; Hieronymus H.; Parker J.; Chang K.; Taron M.; Rosell R.; Moonsamy P.; Dahlman K.; Miller V. A.; Costa C.; Hannon G.; Sawyers C. L. FAS and NF-κB Signalling Modulate Dependence of Lung Cancers on Mutant EGFR. Nature 2011, 471 (7339), 523–526. 10.1038/nature09870. 21430781 PMC3541675

[ref64] Mempel T. R.; Krappmann D. Combining Precision Oncology and Immunotherapy by Targeting the MALT1 Protease. Journal for ImmunoTherapy of Cancer 2022, 10 (10), e005442 10.1136/jitc-2022-005442. 36270731 PMC9594517

[ref65] Araya J.; Cambier S.; Markovics J. A.; Wolters P.; Jablons D.; Hill A.; Finkbeiner W.; Jones K.; Broaddus V. C.; Sheppard D.; Barzcak A.; Xiao Y.; Erle D. J.; Nishimura S. L. Squamous Metaplasia Amplifies Pathologic Epithelial-Mesenchymal Interactions in COPD Patients. J. Clin. Invest. 2007, 117 (11), 3551–3562. 10.1172/JCI32526. 17965775 PMC2040320

[ref66] YOU L.; NA F.; ZHOU J.; JIAO L.; ZHOU Y.; YING B. Expression and Prognosis Analyses of Dectin-1 Cluster Genes in Patients with Lung Adenocarcinoma (LUAD) and the Association with Immune Checkpoint Molecules. BIOCELL 2021, 45 (3), 649–663. 10.32604/biocell.2021.013978.

[ref67] Park M. S.; Yang A.-Y.; Lee J. E.; Kim S. K.; Roe J.; Park M.-S.; Oh M. J.; An H. J.; Kim M.-Y. GALNT3 Suppresses Lung Cancer by Inhibiting Myeloid-Derived Suppressor Cell Infiltration and Angiogenesis in a TNFR and c-MET Pathway-Dependent Manner. Cancer Letters 2021, 521, 294–307. 10.1016/j.canlet.2021.08.015. 34416337

[ref68] Perez-Riverol Y.; Csordas A.; Bai J.; Bernal-Llinares M.; Hewapathirana S.; Kundu D. J.; Inuganti A.; Griss J.; Mayer G.; Eisenacher M.; Pérez E.; Uszkoreit J.; Pfeuffer J.; Sachsenberg T.; Yilmaz S.; Tiwary S.; Cox J.; Audain E.; Walzer M.; Jarnuczak A. F.; Ternent T.; Brazma A.; Vizcaíno J. A. The PRIDE Database and Related Tools and Resources in 2019: Improving Support for Quantification Data. Nucleic Acids Res. 2019, 47 (D1), D442–D450. 10.1093/nar/gky1106. 30395289 PMC6323896

