# Supplementary material for: Comparative Analysis of Transcriptomic and Proteomic Expression between Two Non-Small Cell Lung Cancer Subtypes
Source: J Proteome Res. 2025 Jan 8;24(2):729–41. doi: 10.1021/acs.jproteome.4c00773 (PMC11811994; doi:10.1021/acs.jproteome.4c00773)
Supplement: Supplementary file 1 — pr4c00773_si_001.pdf [file pr4c00773_si_001.pdf]

Supporting Information for Comparative analysis of transcriptomic and proteomic expression between two non-small cell lung cancer subtypes

*#Ben Nicholas[1,2], #Alistair Bailey[1,2], Katy J McCann[3], Peter Johnson[4], Tim Elliott[2,5], Christian Ottensmeier[3,6] and Paul Skipp[1]

[1]Centre for Proteomic Research, Biological Sciences and Institute for Life Sciences, Building 85, University of Southampton, SO17 1BJ UK

[2]Centre for Cancer Immunology and Institute for Life Sciences, Faculty of Medicine, University of Southampton, SO16 6YD UK

[3]School of Cancer Sciences, Faculty of Medicine, University of Southampton, Southampton, SO16 6YD UK

[5]Oxford Cancer Centre for Immuno-Oncology and CAMS-Oxford Institute, Nuffield Department of Medicine, University of Oxford, OX3 7LE UK

[4]Cancer Research UK Clinical Centre, University of Southampton, Southampton, SO16 6YD UK

[6]Institute of Systems, Molecular and Integrative Biology, University of Liverpool, Liverpool, L69 7BE UK

#These authors contributed equally. *Corresponding author

Correspondence to Dr Ben Nicholas. Email: bln1@soton.ac.uk

Contents

Figures S1 to S8 are contained in this document, Tables S1-S17 are csv files. The column names and contents of the csv files are described in this document.

- Section 1 : Figures S1 and S2. PCA bi-plots of NSCLC subtypes PBMC and NAT comparisons
- Section 2 : Figures S3 and S4. Volcano plots of NSCLC subtypes PBMC and NAT comparisons.
- Section 3 : Figures S5 to S7. Heatmaps of NSCLC DEGs and DEPs.
- Section 4 : Figure S8. Bar plots of functional enrichment between NSCLC subtypes and PBMC and NAT.
- Section 5 : Tables S1-3. Gene counts from the HISAT2 alignments estimated by featureCounts and Tables S4-6. Gene counts from transcript classification by Salmon.
- Section 6 : Tables S7-9. Differential gene expression edgeR outputs.
- Section 7 : Tables S10-12. Peaks normalised top 3 peptide intensities.
- Section 8 : Tables S13-15. Differential protein expression DEqMS outputs.
- Section 9 : Tables S16-17. Functional enrichment analysis g:Profiler outputs.

PCA bi-plots of NSCLC subtypes PBMC and NAT comparisons

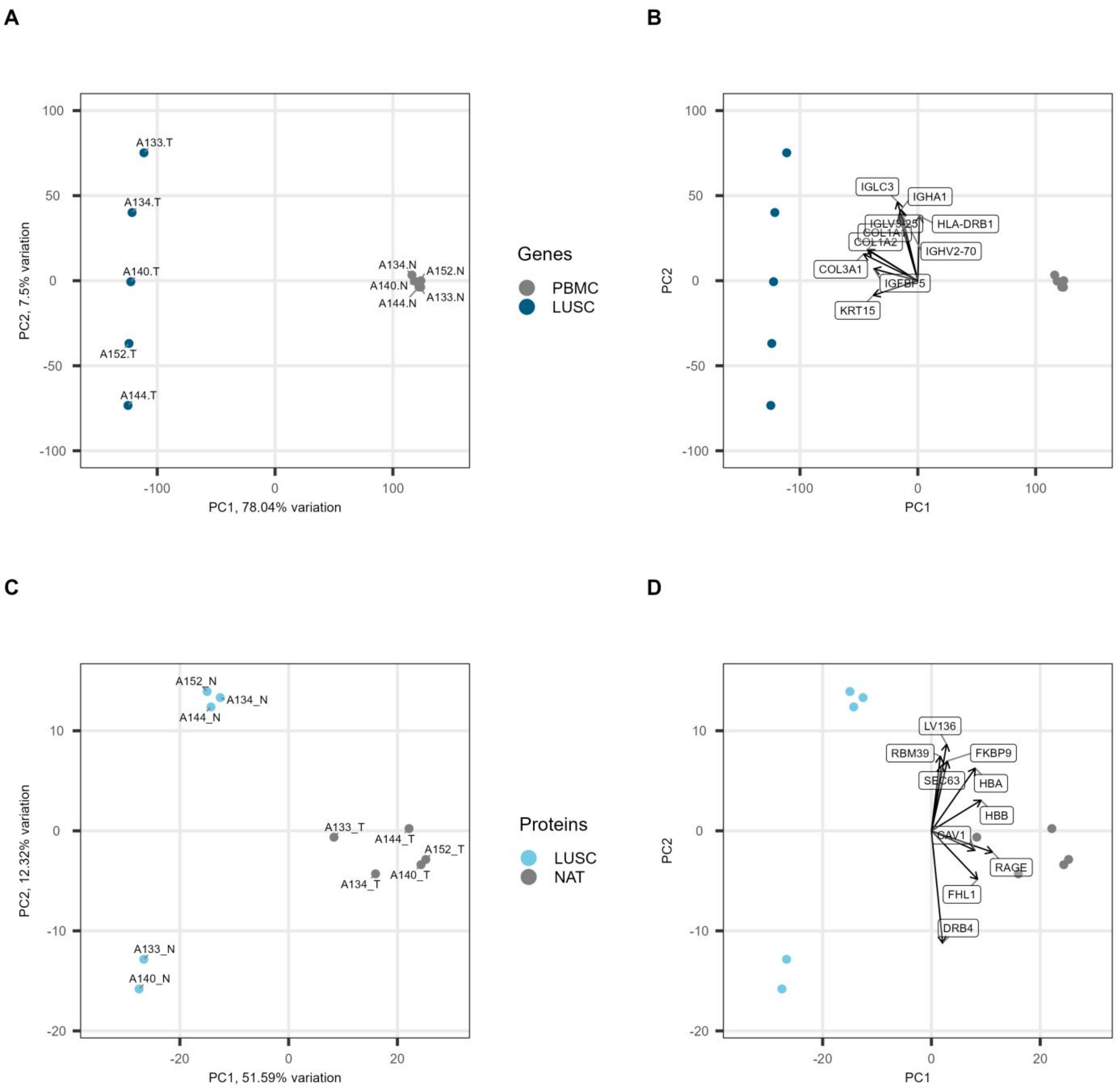

Figure S1: Bi-plots of the LUSC and PBMC and NAT comparison. (A) PCA of normalised gene count matrix numbered with donor identifier. LUSC (blue) & PBMC (grey). (B) PCA of normalised gene count matrix with the genes contributing to the PC

directions annotated. (C) PCA of normalised top 3 peptide intensities numbered with donor identifier. LUSC (light blue) & NAT (grey). (D) PCA of normalised top 3 peptide intensities with the protein contributing to the PC directions annotated.

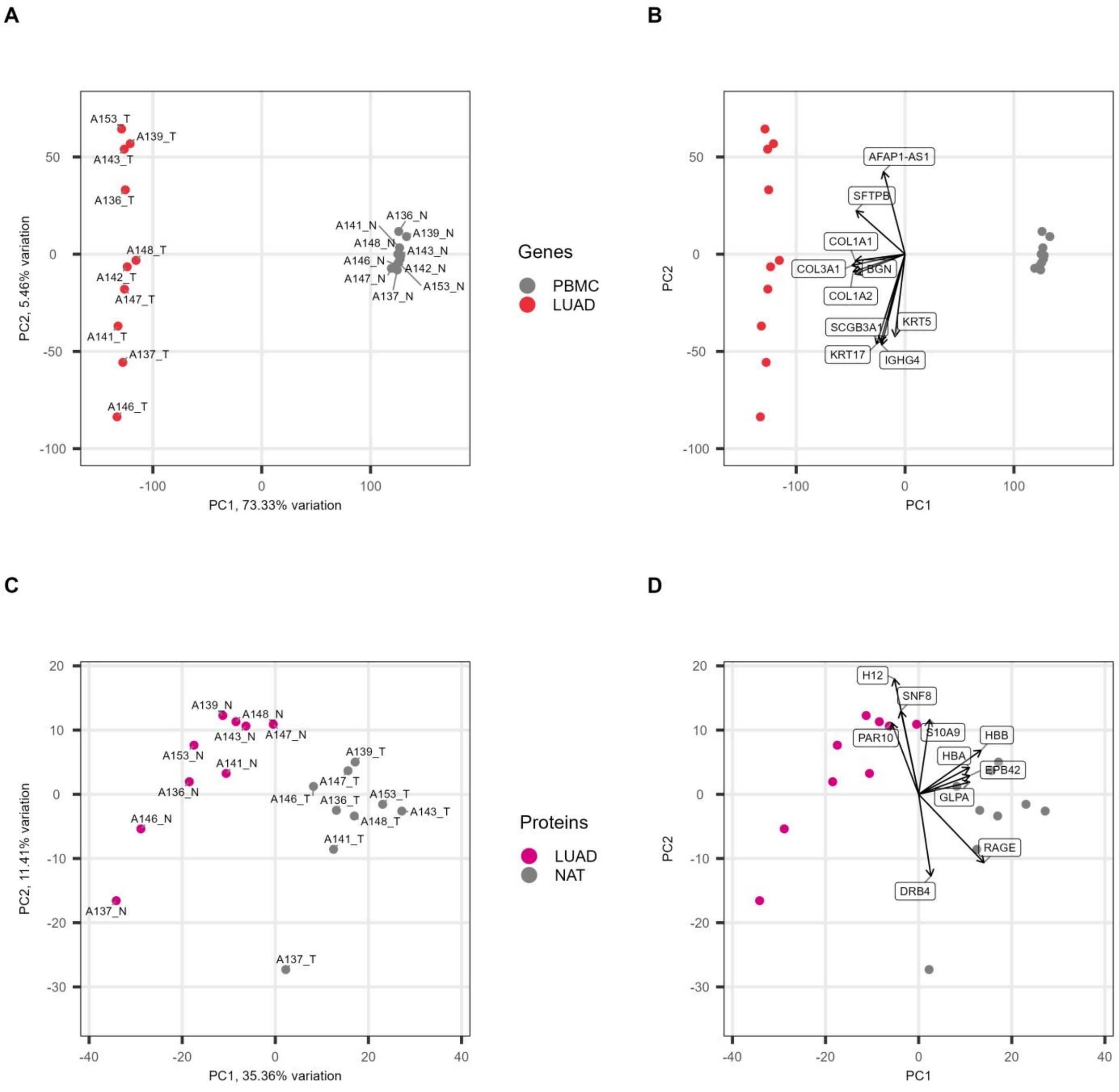

Figure S2: Bi-plots of the LUAD and PBMC and NAT comparison. (A) PCA of normalised gene count matrix numbered with donor identifier. LUAD (red) & PBMC

(grey). (B) PCA of normalised gene count matrix with the genes contributing to the PC directions annotated. (C) PCA of normalised top 3 peptide intensities numbered with donor identifier. LUAD (purple) & NAT (grey). (D) PCA of normalised top 3 peptide intensities with the protein contributing to the PC directions annotated.

Volcano plots of NSCLC subtypes PBMC and NAT comparisons

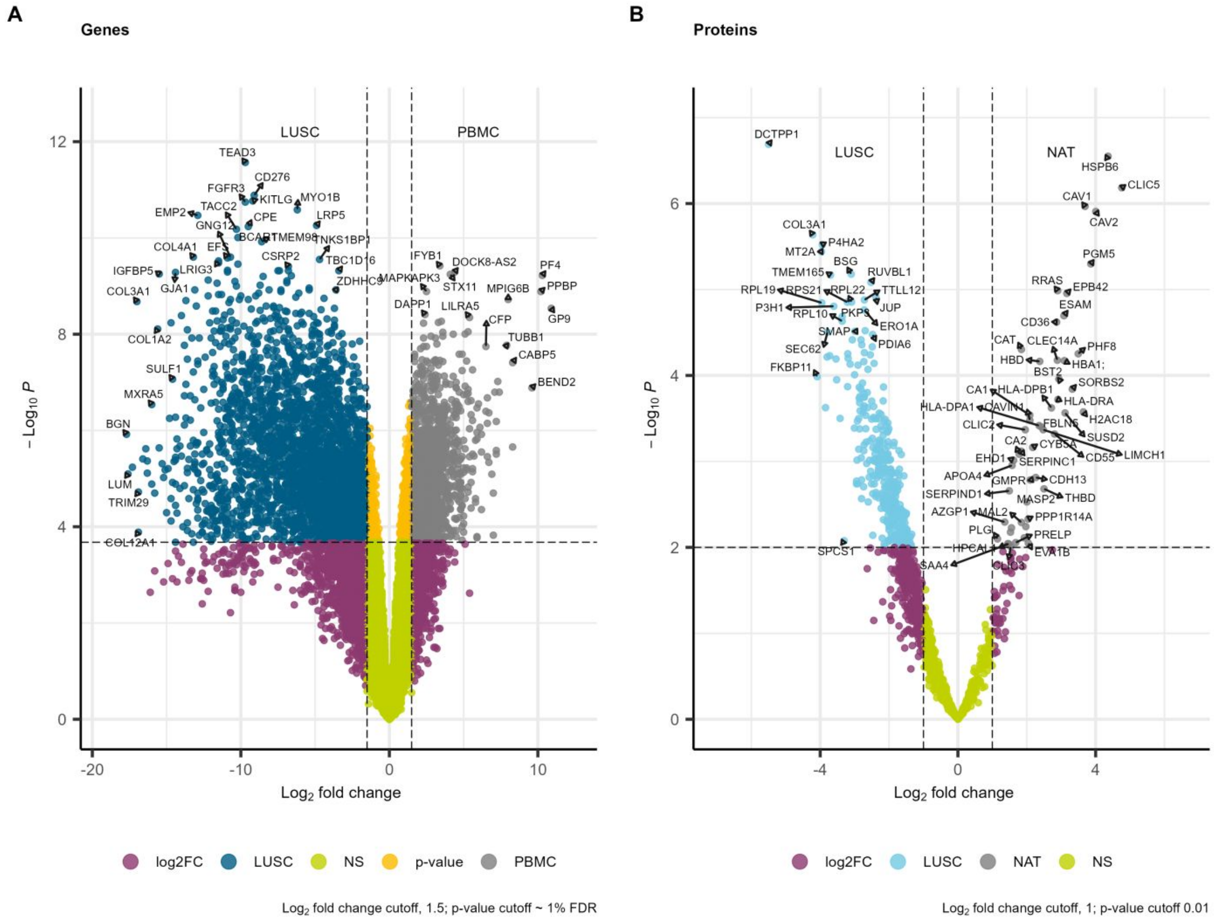

Figure S3: (A) Comparison of LUSC & PBMC (n=17,719). Thresholds are represented by dotted lines at FDR of 1% and log_2 fold change of 1.5. (B) Comparison of LUSC & NAT

(n=1,330). Thresholds are represented by dotted lines at p-value of 1% and log$_2$ fold change of 1.

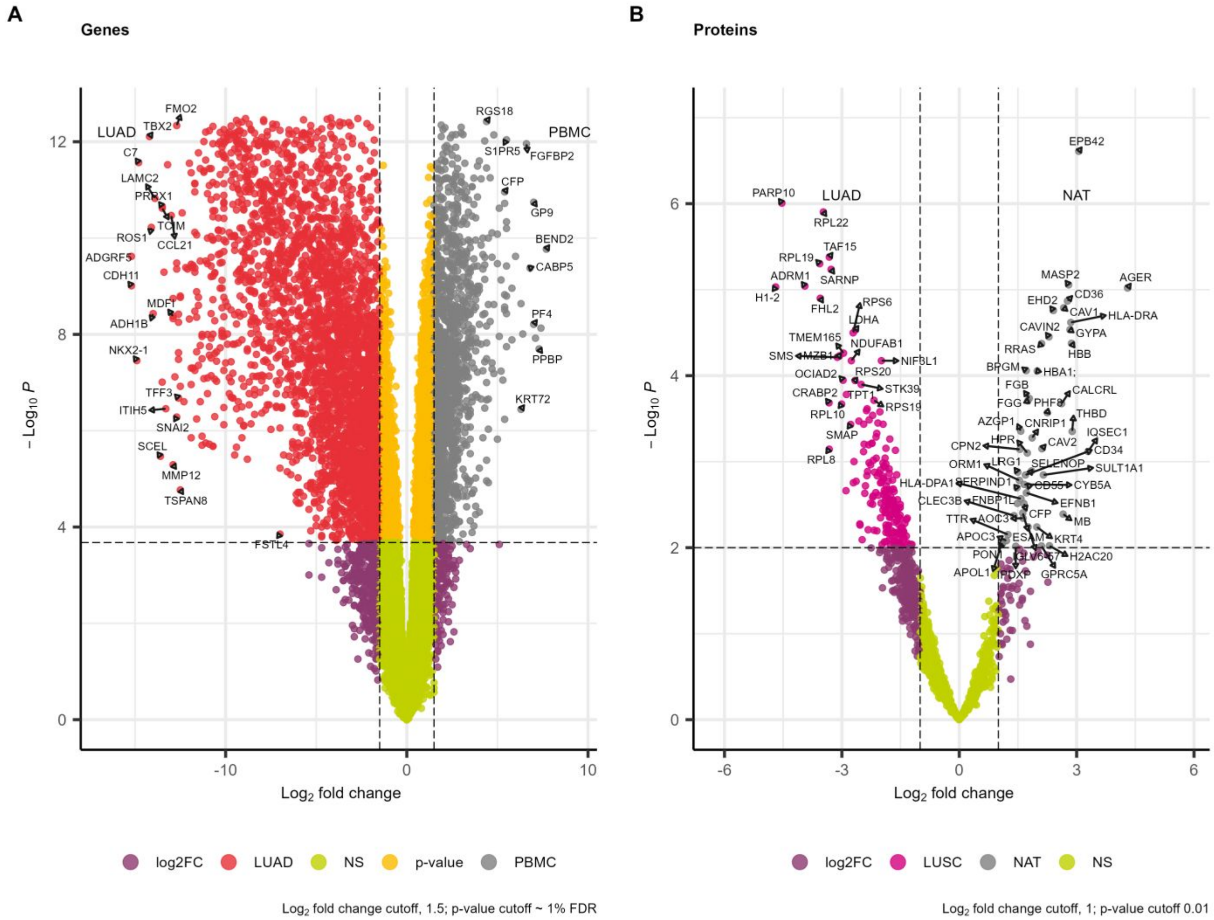

Figure S4: (A) Comparison of LUAD & PBMC (n=17,586). Thresholds are represented by dotted lines at FDR of 1% and log$_2$ fold change of 1.5. (B) Comparison of LUAD & NAT (n=1,478). Thresholds are represented by dotted lines at p-value of 1% and log$_2$ fold change of 1.

Heatmaps of NSCLC DEGs and DEPs

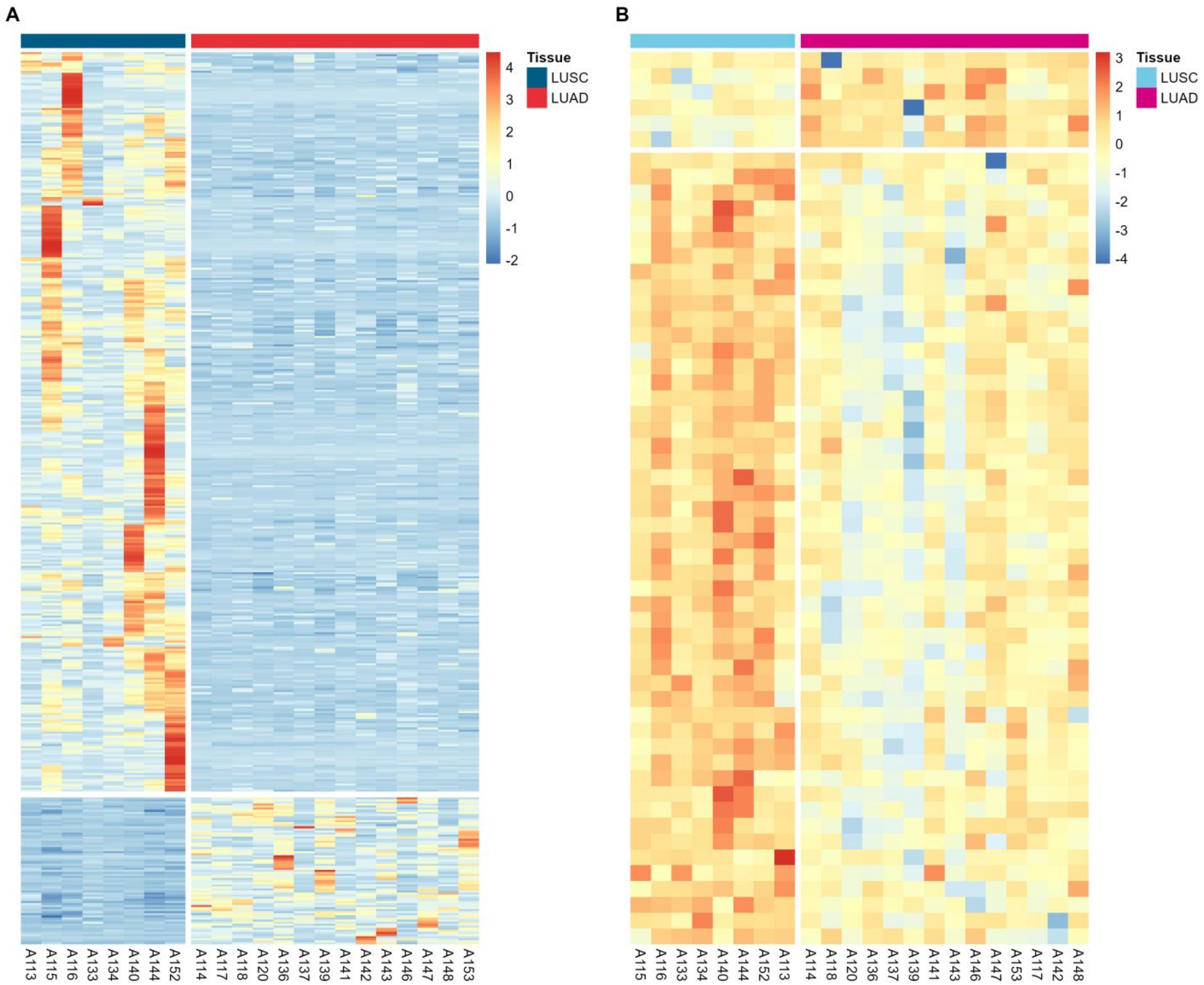

Figure S5: (A) Comparison of LUSC & LUAD DEGs below a FDR of 1%. (n=428). (B) Comparison of LUSC & LUAD DEPs below a p-value 1% (n=139). Colour bar shows $\log_2$ fold change rescaled as z-scores i.e. each unit from zero represents one standard deviation from the row average value for each protein.

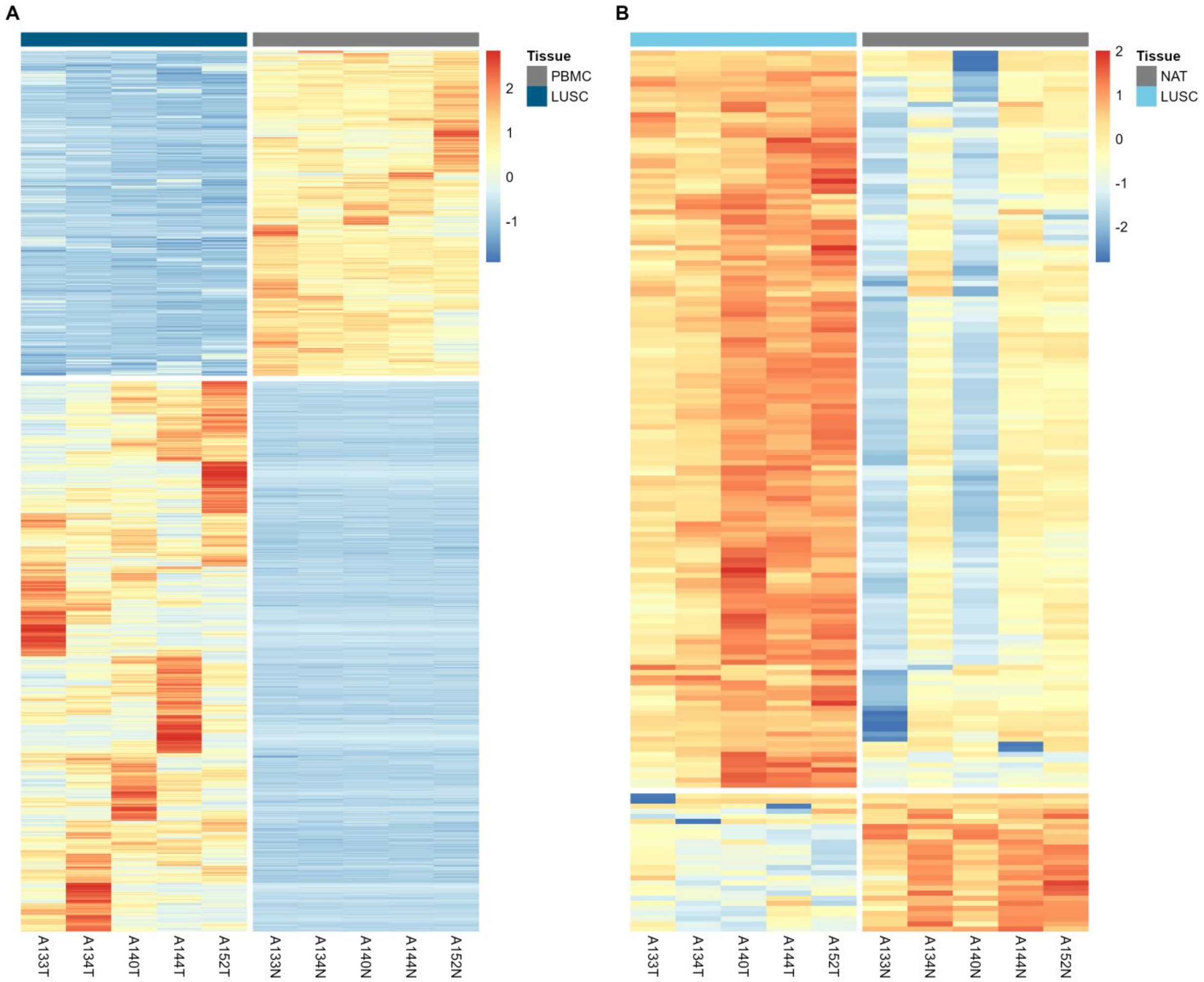

Figure S6: (A) Comparison of LUSC & PBMC DEGs below a FDR of 1%. (n=8,089). (B) Comparison of LUSC & NAT DEPs below a p-value 1% (n=379). Colour bar shows log_2 fold change rescaled as z-scores i.e. each unit from zero represents one standard deviation from the row average value for each protein.

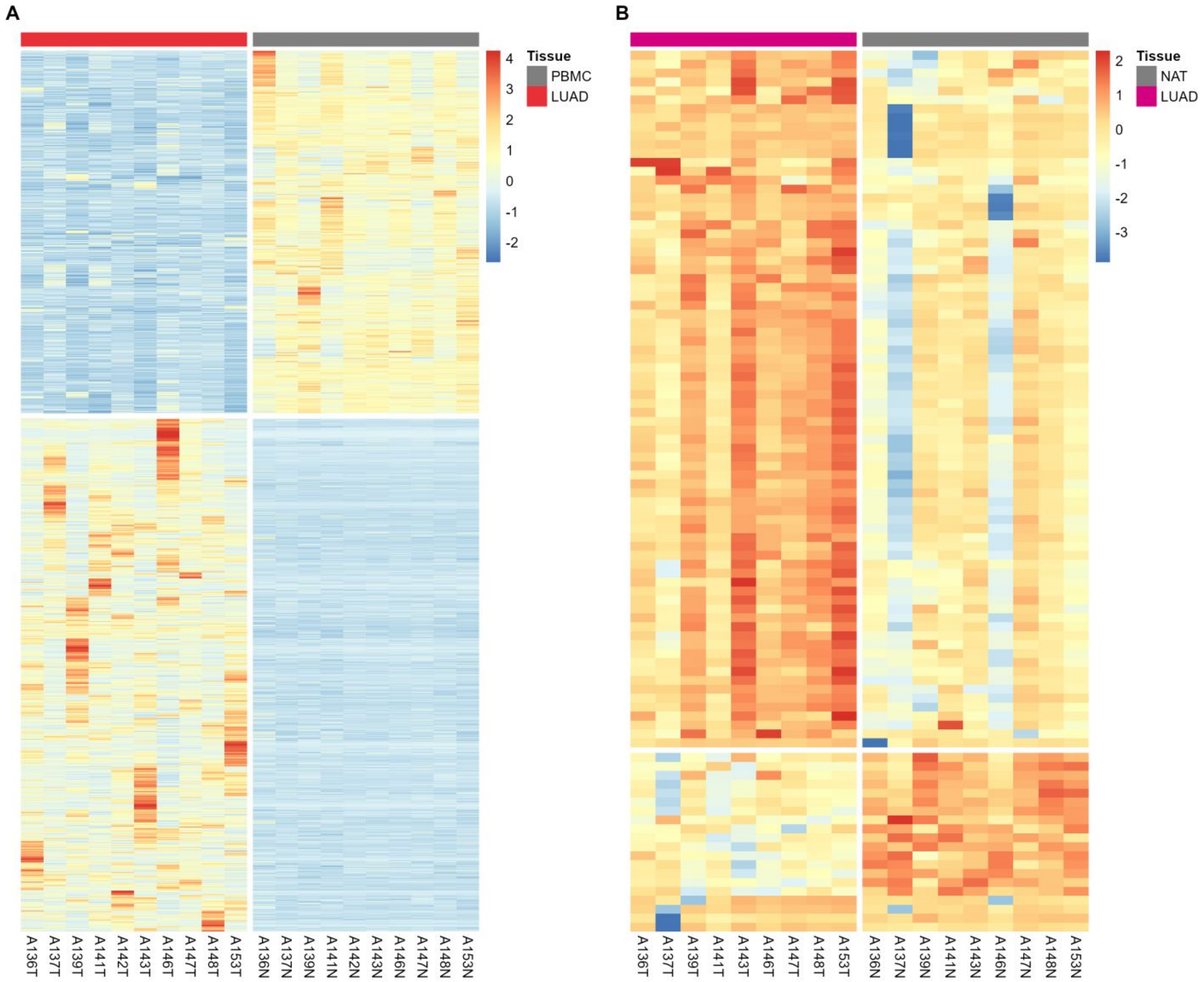

Figure S7: (A) Comparison of LUAD & PBMC DEGs below a FDR of 1%. (n=10,058). (B) Comparison of LUAD & NAT DEPs below a p-value 1% (n=234). Colour bar shows $\log_2$ fold change rescaled as z-scores i.e. each unit from zero represents one standard deviation from the row average value for each protein.

Functional analysis of NSCLC subtpyes PBMC and NAT comparisons

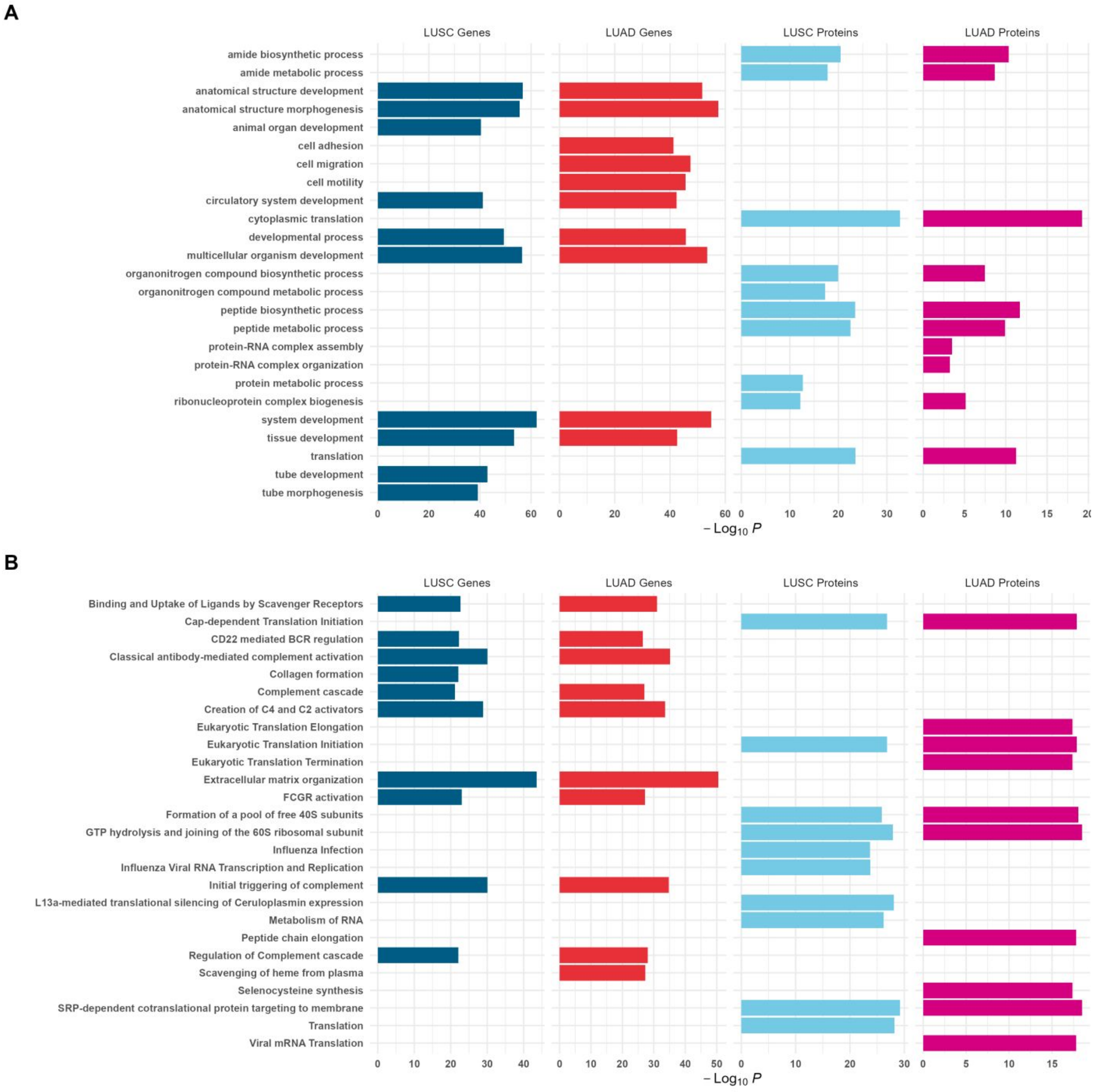

Figure S8: Bar plots of functional enrichment between NSCLC subtypes and PBMC and NAT. Statistical significance level indicated by the -log$_{10}$ p-value on the x-axis. (A) GO

biological processes enriched in NSCLC subtypes. (B) Reactome pathways enriched in NSCLC subtypes.

Transcript quantification and gene counts

Transcripts were quantified by genomic alignments using HISAT2 (version 2.2.1)[1] and featureCounts (version 2.0.6)[2], and by transcript classification using Salmon (version 1.10.3)[3].

HISAT2

Tables S1-3 contain the gene counts from the HISAT2 alignments estimated by featureCounts.

Table 1

File
Table-S1-Hisat-LUAD-vs-PBMC-counts.csv
Table-S2-Hisat-LUSC-vs-PBMC-counts.csv
Table-S3-Hisat-LUSC-vs-LUAD-counts.csv

HISAT2 Counts Tables Information

Column name	Description
`name`	Ensembl gene identifier
`gene`	HGNC gene symbol
`sample_id` the donor id or donor id suffixed with `T` for tumour or `N` for PBMC samples.	mapped read counts from featureCounts

Salmon

Tables S4-6 contain the gene counts from transcript classification by Salmon.

Table 2

File
Table-S4-Salmon-LUAD-vs-PBMC-counts.csv
Table-S5-Salmon-LUSC-vs-PBMC-counts.csv
Table-S6-Salmon-LUSC-vs-LUAD-counts.csv

Salmon Counts Table Information

Column name	Description
`name`	Ensembl gene identifier
`gene`	HGNC gene symbol or Ensembl if missing
`length`	the length of the target transcript
`sample_id` the donor id or donor id suffixed with `T` for tumour or `N` for PBMC samples.	mapped reads counts by Salmon

Differential gene expression with EdgeR

Differential gene expression (DEG) was estimated using EdgeR and default settings[4]. Results were filtered for common DEG from both HISAT2 and Salmon counts

Tables S7-9 contain the edgeR outputs.

Table 3

File
Table-S7-edgeR-DEG-LUAD-vs-PBMC.csv
Table-S8-edgeR-DEG-LUSC-vs-PBMC.csv
Table-S9-edgeR-DEG-LUSC-vs-LUAD.csv

edgeR Table information

Column name	Description
`name`	Ensembl gene identifier
`gene`	HGNC gene symbol or Ensembl if missing
`baseMean`	mean read counts
`baseMeanA`	mean read count group A
`baseMeanB`	mean read count group B
`foldChange`	fold change B/A
`log2FoldChange`	log2 fold change B/A
`PValue`	p-value
`PAdj`	Benjamini-Hochbergadjusted p-value
`FDR`	False discovery rate
`falsePos`	false discovery counts
`sample_id` the donor id or donor id suffixed with `T` for tumour or `N` for PBMC samples.	sample HISAT2 read count

Peaks normalised Top 3 peptide intensities

Label free quantification using the Peaks Q module of Peaks Studio[5,6] yielding matrices of protein identifications as quantified by their normalised top 3 peptide intensities.

Tables S10-12 contain normalised top 3 peptide intensities.

Table 4

File
Table-S10-Peaks-top3-peptides-intensities-LUAD-vs-NAT.csv
Table-S11-Peaks-top3-peptides-intensities-LUSC-vs-NAT.csv
Table-S12-Peaks-top3-peptides-intensities-LUSC-vs-LUAD.csv

Peaks normalised Top 3 peptide intensities Table information

Column name	Description
`protein`	protein short name
`gene`	HGNC gene symbol
`sample_id` the donor id or donor id suffixed with `T` for tumour or `N` for NAT samples	Normalised top 3 peptide intensity from Peaks

Differential protein expression with DEqMS

The normalised top 3 peptide intensities were filtered to remove any proteins for which there were more than two missing values across the samples. Differential protein expression (DEP) was then calculated with DEqMS using the default steps[7].

Tables S13-15 contain the outputs of DEqMS.

Table 5

**File**
Table-S13-DEqMS-DEP-LUAD-vs-NAT.csv
Table-S14-DEqMS-DEP-LUSC-vs-NAT.csv
Table-S15-DEqMS-DEP-LUSC-vs-LUAD.csv

DEqMS Table information

Column name	Description
`logFC`	log2 fold change between two groups
`AveExpr`	the mean of the log2 ratios across all samples
`t`	Limma t-values
`P.Value`	Limma p-values
`adj.P.Val`	BH method adjusted Limma p-values
`B`	Limma B values
`gene`	HGNC gene symbol
`count`	peptide count values
`sca.t`	DEqMS t-statistics
`sca.P.Value`	DEqMS p-values
`sca.adj.pval`	BH method adjusted DEqMS p-values
`protein`	protein short name

Functional analysis with g:Profiler

Functional enrichment analysis used g:Profiler[8] using G:OSt `multi_query` in default settings for homo sapiens modified to exclude GO electronic annotations. Gene ids were used as inputs for DEGs and protein ids for DEPs.

We used four lists as inputs to the multiple query setting for G:OSt for comparing NSCLC subtypes for DEGs filtered at thresholds 5% FDR and logFC 1.5 and DEPs filtered p-val 5% and logFC 1 for protein expression. The identifiers for each list are `LUAD Genes`: LUAD DEGs, `LUAD Proteins`: LUAD DEPs, `LUSC Genes` : LUSC DEGs, `LUSC Proteins`: LUSC DEPs.

The four lists for comparison of NSCLC subtypes with PBMC were NSCLC DEGs filtered at 1% FDR and logFC 1.5 and NSCLC DEPs 5% FDR and logFC of 1 for NAT comparison. The list identifiers are as for NSCLC subtype comparison.

Table S16 contain the g:Profiler outputs for the NSCLC comparisons and Table S17 the NSCLC and PBMC/NAT comparisons.

Table 6

**File**
Table-S16-gprofiler-LUSC-vs-LUAD.csv
Table-S17-gprofiler-NSCLC-vs-NORM.csv

g:Profiler Table information

Column name	Description
`term_id`	unique term identifier

Column name	Description
p_values	hypergeometric p-value after correction for multiple testing
significant	indicator for statistically significant results
term_size	number of genes that are annotated to the term
query_sizes	number of genes that were included in the query
intersection_sizes	the number of genes in the input query that are annotated to the corresponding term
source	the abbreviation of the data source for the term (e.g. GO:BP)
term_name	the ontology term name
effective_domain_size	the total number of genes "in the universe" used for the hypergeometric test
source_order	numeric order for the term within its data source
parents	list of term IDs that are hierarchically directly above the term. For non-hierarchical data sources this points to an artificial root node.
id	The identifier of for the input list associated with the row e.g. LUAD Genes were the list of LUAD DEGs.

On-line Supporting Information

An online version of the Supporting Information is also available on Github: https://github.com/ab604/lung-global-supplement associated with Zenodo DOI: https://zenodo.org/doi/10.5281/zenodo.13327662.

References

(1) Kim, D.; Paggi, J. M.; Park, C.; Bennett, C.; Salzberg, S. L. Graph-Based Genome Alignment and Genotyping with HISAT2 and HISAT-Genotype. *Nature Biotechnology* **2019**, *37* (8), 907–915. https://doi.org/10.1038/s41587-019-0201-4.

(2) Liao, Y.; Smyth, G. K.; Shi, W. featureCounts: An Efficient General Purpose Program for Assigning Sequence Reads to Genomic Features. *Bioinformatics* **2013**, *30* (7), 923–930. https://doi.org/10.1093/bioinformatics/btt656.

(3) Srivastava, A.; Malik, L.; Sarkar, H.; Patro, R. A Bayesian Framework for Inter-Cellular Information Sharing Improves dscRNA-Seq Quantification. *Bioinformatics* **2020**, *36* (Supplement_1), i292–i299. https://doi.org/10.1093/bioinformatics/btaa450.

(4) Yunshun Chen , Aaron Lun, Davis McCarthy , Xiaobei Zhou , Mark Robinson, Gordon Smyth. edgeR. **2017**. https://doi.org/10.18129/B9.BIOC.EDGER.

(5) Zhang, J.; Xin, L.; Shan, B.; Chen, W.; Xie, M.; Yuen, D.; Zhang, W.; Zhang, Z.; Lajoie, G. A.; Ma, B. PEAKS DB: De Novo Sequencing Assisted Database Search for Sensitive and Accurate Peptide Identification. *Molecular & Cellular Proteomics* **2012**, *11* (4), M111010587.

(6) Lin, H.; He, L.; Ma, B. A Combinatorial Approach to the Peptide Feature Matching Problem for Label-Free Quantification. *Bioinformatics* **2013**, *29* (14), 1768–1775. https://doi.org/10.1093/bioinformatics/btt274.

(7) DEqMS. http://bioconductor.org/packages/DEqMS/.

(8) Kolberg, L.; Raudvere, U.; Kuzmin, I.; Vilo, J.; Peterson, H. Gprofiler2 – an R Package for Gene List Functional Enrichment Analysis and Namespace Conversion Toolset g:Profiler. *F1000Research* **2020**, *9*, 709. https://doi.org/10.12688/f1000research.24956.2.
